# Broad-spectrum antifungal activity of C12/C14 alkyl triphenylphosphonium salts (TPP-C_12_ and TPP-C_14_) against clinically relevant pathogens

**DOI:** 10.3389/fmicb.2025.1693528

**Published:** 2026-01-26

**Authors:** Yuanyuan Geng, Xiaohui Wang, Shu Zhang, Xuelian Liu, Huihui Liu, Xiaonan Guo, Yangzhen Lu, Jie Gong, Zhaohai Qin

**Affiliations:** 1 National Key Laboratory of Intelligent Tracking and Forecasting for Infectious Diseases, National Institute for Communicable Disease Control and Prevention, Chinese Center for Disease Control and Prevention, Beijing, China; 2 National Institute for Communicable Disease Control and Prevention Joint Laboratory of Pathogenic Fungi, Peking University First Hospital, Beijing, China; 3 Department of Gastroenterology, The Sixth Medical Center, Chinese PLA General Hospital, Beijing, China; 4 College of Science, China Agricultural University, Beijing, China; 5 Collaborative Innovation Center of Recovery and Reconstruction of Degraded Ecosystem in Wanjiang Basin Co-Founded by Anhui Province and Ministry of Education, School of Ecology and Environment, Anhui Normal University, Wuhu, China

**Keywords:** antifungal activity, antifungal mechanism, pathogenic fungi, transcriptome, triphenylphosphonium

## Abstract

**Introduction:**

Human fungal infections affect billions of people and result in more than 2 million deaths every year, however, they have historically been neglected as a cause of infectious disease-related deaths worldwide. Fungal drug resistance has become an increasingly serious problem with the wide use of antifungal drugs and the adaptive evolution of fungi. Resistance to all commonly used antifungal drugs has been reported, and the development of non-traditional antifungal drugs is urgently needed.

**Methods:**

Minimal inhibitory concentrations (MICs) of clinical pathogenic fungi were assessed by broth dilution antifungal susceptibility testing. One hundred and twenty eight yeast strains and 66 filamentous strains were used, including *C. albicans* resistant and susceptible to azoles, *C. tropicalis, C. auris, C. krusei*, the *C. glabrata* complex, the *C. haemulonii* complex, the *C. parapsilosis* complex, *Cryptococcus neoformans, Aspergillum, Trichophyton*, and dimorphic *Sporothrix globosa*. Further RNAseq was performed to explore the antifungal mechanism of two derivatives.

**Results:**

Two derivatives of the mitochondrion-targeted compound triphenylphosphonium (TPP), TPP-C_12_ and TPP-C_14_, showed broad-spectrum antifungal activity. The MIC against yeast strains was 1.5173 and 1.0109 mg/L, respectively. For filamentous strains, the MIC ranges were 2–8 mg/L for both compounds. For the dimorphic *Sporothrix globosa*, the GM values were 1.0134 and 1.0816 mg/L, respectively. RNAseq revealed that the derivatives interfered with mainly mitochondrial and ribosomal functions. Through coregulation of mitochondrial and nuclear genes, the derivatives cause mitochondrial dysfunction and ultimately cell death.

**Discussion:**

Taken together, the findings show that TPP-C_12_ and TPP-C_14_ are stable, effective, and broad-spectrum antifungal agents with no species or strain specificity.

## 1 Introduction

In recent years, the occurrence of fungal infections has become a significant health concern, with updated estimates suggesting an annual incidence of 6.5 million invasive fungal infections and 3.8 million associated deaths (Denning, 2024). The main pathogens causing invasive fungal infections include *Candida, Aspergillus*, and *Cryptococcus* (Köhler et al., 2017; Brown et al., 2024; Denning, 2024). With respect to superficial fungal infection, species within the *Trichophyton rubrum* complex are the dominant pathogens, accounting for more than 80% of dermatophyte infections (Geng et al., 2021; Dellière et al., 2024). Besides, the proportion of *T. mentagrophytes* is gradually increasing, including *T. indotineae*, a prominent genotype linked to widespread, severe, and terbinafine-resistant infections (Shao et al., 2025). Pathogenic fungal infections pose a significant threat to public health and safety.

The types of antifungal drugs historically used in clinical practice to treat fungal infections include mainly azoles (such as fluconazole, itraconazole, posaconazole, and voriconazole), echinocandins (such as anidulafungin, caspofungin, and micafungin), polyenes (such as amphotericin B), and pyrimidine analogs (such as 5-fluorocytosine; Fisher et al., 2022). In recent years, with the widespread use of antifungal chemicals (in both humans and crops) and the rapid adaptive evolution of fungi, fungal resistance has become an increasingly serious problem, especially resistance against azoles. The evolving resistance of clinical fungal pathogens to all licensed systemic antifungal drugs exacerbates the difficulty of treating fungal infections (Perlin et al., 2017; Fisher et al., 2018, 2022).

Triphenylphosphonium (TPP) is a lipophilic cation that consists of a positively charged phosphorus atom surrounded by three hydrophobic phenyl groups, which provide an extended hydrophobic surface despite the positive charge of the phosphorus atom. Owing to its structural advantages, TPP can achieve efficient uptake and accumulation in mitochondria (Zielonka et al., 2017), and has been widely used in the design of mitochondrion-targeted compounds, including anticancer, antifungal, antiparasitic, and antioxidant compounds (Wang et al., 2020).

In the present study, the antifungal activities of two alkyl-TPP derivatives were investigated against a range of clinical fungal species. Transcriptomic analysis after TPP derivative treatment was performed to explore the potential functional mechanism of these compounds.

## 2 Materials and methods

### 2.1 Fungal strains and media

A total of 128 yeast isolates were used in this study, including *Candida albicans* (*n* = 18; 8 strains were resistant to azoles, while the remaining 10 isolates were susceptible), *C. tropicalis* (*n* = 10), *C. auris* (*n* = 3), *C. bracarensis* (*n* = 3), *C. glabrata* (*n* = 10), *C. krusei* (*n* = 10), *C. metapsilosis* (*n* = 10), *C. nivariensis* (*n* = 10), *C. orthopsilosis* (*n* = 10), *C. parapsilosis* (*n* = 10), *C. haemulonii* complex (10 × *C. haemolnii*, 4 × *C. haemulonis* var. *vulnera* (*n* = 4), 10 × *C. duobushaemulonii*), and *Cryptococcus neoformans* (*n* = 10). In addition, 5 strains of *Aspergillum* spp. (*A, flavus, A. niger, A. terreus, A. nidulans, A. fumigatus*), 6 strains of dermatophytes (3 *Trichophyton rubrum*, 2 *T. mentagrophytes*, and 1 *T. soudanense*) and 55 dimorphic *Sporothrix globosa* strains were also selected for the experiments. *Candida parapsilosis* (ATCC 22019) and *Candida krusei* (ATCC 6258) were used for quality control. All strains were collected and stored by National Institute for Communicable Disease Control and Prevention, Chinese Center for Disease Control and Prevention.

All strains were subcultured twice by inoculation onto Sabouraud dextrose agar (SDA) medium (Land Bridge, Beijing, China) supplemented with 0.05% chloramphenicol (Sangon Biotech, Shanghai, China) at 28 °C. All the strains were identified by morphological observation and amplification of the internal transcribed spacer (ITS) region. The primer sequences were as follows:

ITS1 (forward): 5′-TCCGTAGGTGAACCTGCGG-3′;

ITS4 (reverse): 5′-TCCTCCGCTTATTGATATGC-3′ (White et al., 1990).

Alignment of the ITS sequence was performed using National Center for Biotechnology Information (NCBI) nucleotide basic local alignment search tool (BLAST). The sequencing data were submitted to GenBank, and accession numbers were listed in Supplementary Table S1.

Roswell Park Memorial Institute 1640 (RPMI-1640; Gibco, New York, USA) culture medium supplemented with 2% glucose and 0.165 M morpholinepropanesulfonic acid (MOPS; final pH adjusted to 7.0) was used for antifungal susceptibility testing.

### 2.2 Antifungal agents and chemicals

**Figure 1:**
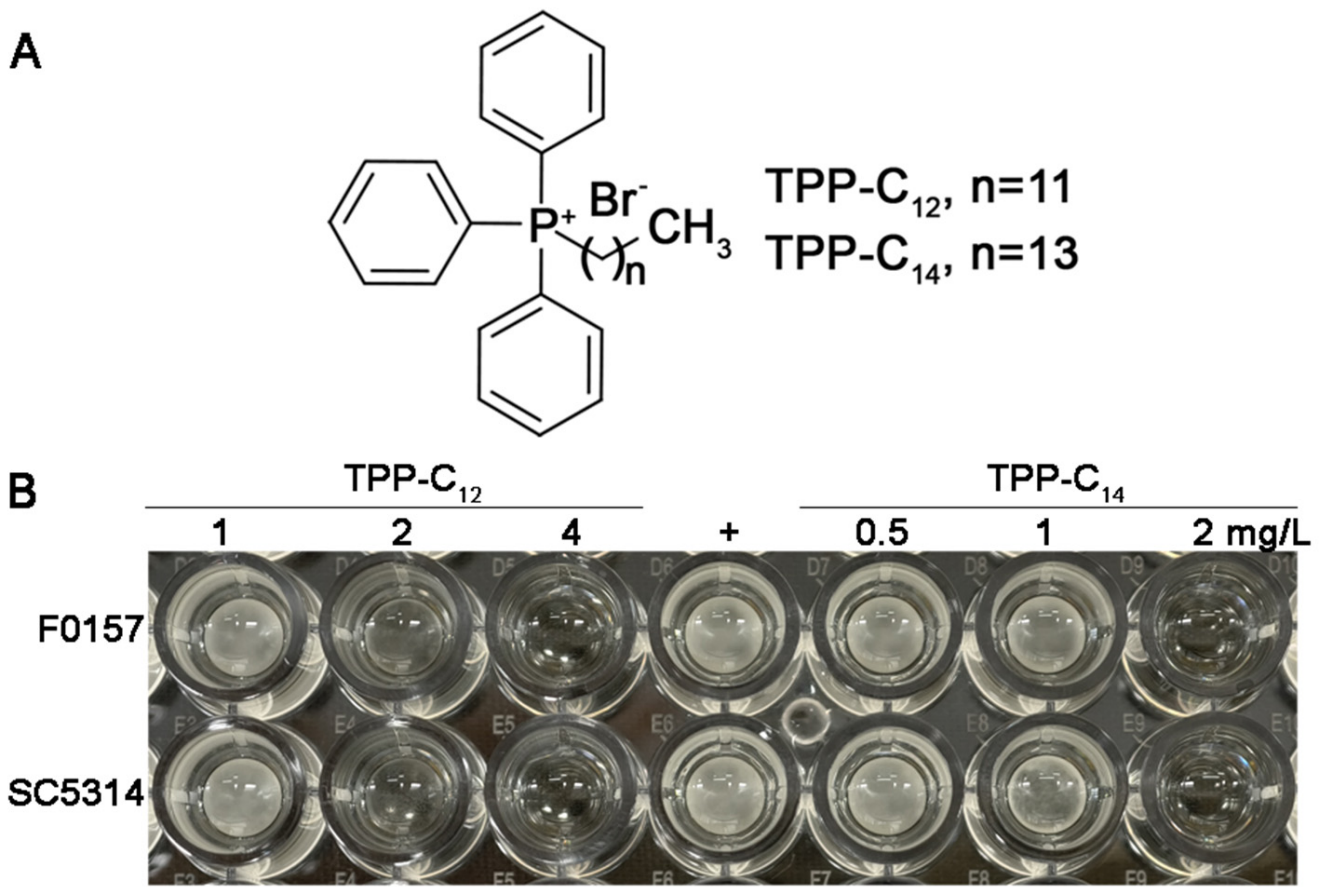
Chemical structures and antifungal activity of two TPP- derivatives. **(A)** The figure shows the structure of dodecyl triphenylphosphonium bromide (TPP-C_12_) and tetradecyl triphenylphosphonium bromide (TPP-C_14_). **(B)** The inhibitory effect of TPP- derivatives on the growth of azole-resistant *C. albicans* F0157 and azole-susceptible *C. albicans* SC5314. 100 μL *C. albicans* at a McFarland turbidity of 0.5 were incubated with TPP-C_12_ and TPP-C_14_ for 24 h; the final drug concentrations are indicated in the figure. “+” denotes the positive control; no drug is added, and SDB is used as the replacement.

Fluconazole, voriconazole, itraconazole and posaconazole were purchased from Harveybio (Beijing) Gene Technology Co., Ltd. Dodecyl triphenylphosphonium bromide (TPP-C_12_) and tetradecyl triphenylphosphonium bromide (TPP-C_14_) were synthesized by the Qin laboratory (Figure 1A). Serial stock solutions of 100 × final testing concentrations were prepared by dissolving the reagent powder in dimethyl sulfoxide (DMSO).

### 2.3 *In vitro* antifungal susceptibility

A 1:50 dilution with RPMI-1640 medium was performed for the 100 × stock solutions of both antifungal agents, resulting in two-fold working solutions. One hundred microliters of the two-fold working solutions were added to the wells of 96-well plates.

For the yeast isolates, antifungal susceptibility testing was performed according to the CLSI M27 protocol (CLSI, 2017b). In brief, the strains were cultivated in SDA at 28 °C for 24–48 h, followed by suspension in 0.85% sterile saline. For filamentous strains, antifungal susceptibility was assessed according to the CLSI M38 document (CLSI, 2017a). Filamentous strains, including mycelia from dimorphic fungi, were cultivated on potato dextrose agar (PDA; Land Bridge, Beijing, China) for 5–7 days for sporulation. For dermatophytes, the cultivation time was increased to 2 weeks. Conidial suspensions were prepared by scraping the culture surface with a sterile swab and then suspending the cells in 0.85% sterile saline.

The densities of the fungal suspensions at 530 nm were measured with a spectrophotometer and adjusted to 0.15–0.2. A 1:1,000 dilution of the yeast suspensions and a 1:50 dilution of the filamentous conidial suspensions were performed with RPMI-1640 medium to obtain a two-fold dilution as an inoculum. One hundred microliters of diluted inoculum was added to the wells of the 96-well plates containing 100 μL of the two-fold antifungal agents to achieve a 1:1 ratio in the desired final inoculum. The growth control consisted of 100 μL of drug-free RPMI-1640 medium and 100 μL of inoculum. The cells were cultured in 200 μL of drug-free RPMI-1640 medium as a sterility control.

Minimum inhibitory concentrations (MICs) were determined at 24 h for *Candida* spp. and at 72 h for *Cryptococcus neoformans*. For filamentous strains, MICs were determined at 48 h; for dermatophytes, the time was increased to 4 days. In this study, the MICs for TTPP and PTPP were defined as the lowest concentration that achieved 100% inhibition of control growth for all the isolates. The MICs of azoles against yeast and filamentous strains were interpreted according to the definitions of CLSM M27 and M38, respectively. Geometric mean (GM) MICs were calculated where at least 8 strains of a species were tested. All the experiments were carried out in triplicate.

### 2.4 *In vitro* synergy of chemicals and azoles

The fractional inhibitory concentration index (FICI) was used to assess interactions between TPP-C_12_ or TPP-C_14_ and azole and was measured by a checkerboard assay (Halliday et al., 2023). In brief, 50 μL two-fold fluconazole serial dilutions were applied row-wise to the wells of a 96-well plate containing 100 μL of prepared inoculum suspension, after which 50 μL two-fold TPP-C_12_ or TPP-C_14_ serial dilutions were applied column-wise to the wells of the same plate. The results were then analyzed after a 24 h incubation at 28 °C. The FICI was defined as (MIC combined/MIC drug A alone) + (MIC combined/MIC drug B alone). The interaction was considered synergistic when the FICI was ≤ 0.5; no interaction was considered to occur when 0.5 < FICI ≤ 4; and the interaction was considered antagonistic when the FICI was >4. The experiments were conducted in triplicate.

### 2.5 *In vitro* toxicity assays

The toxicity of TPP-C_12_ and TPP-C_14_ was determined by incubating agents with AGS cells, after which cell viability was measured via a Cell Counting Kit-8 (CCK-8; Beyotime Biotechnology, Shanghai, China) according to the manufacturer's instructions. The final concentrations of TTPP were 4, 2, 1, 0.5, and 0.25 μg/mL, while PTPP was added at final concentrations of 8, 4, 2, 1, and 0.5 μg/mL. Cells were incubated with drugs or DMSO for 12, 24, 36, or 48 h (Chen et al., 2024). Drug-free RPMI-1640 with an equivalent volume of DMSO was used as a control. The cytotoxicity of TPP-C_12_ and TPP-C_14_ was evaluated by comparing cell viability with that of the control group. Each treatment was performed in quintuplicate wells, and the experiments were conducted in triplicate.

### 2.6 Mitochondrial assays

The cells were grown overnight in Sabouraud's dextrose broth (SDB; Land Bridge, Beijing, China) with shaking at 28 °C and 180 rpm. Inocula of *C. albicans* were diluted with SDB to an OD600 of 0.1. A total of 40 μL of inoculum was added to 4 mL of fresh SDB for 12 h of cultivation with an orbital shaker at 28 °C and 180 rpm. The cells were then exposed to DMSO or drug (final concentration of 1 μg/mL) for 2 h with shaking. After incubation with the drug, the cells were centrifuged at 3,900 rpm for 10 min and washed with 1 mL of PBS (pH 7.4). Then, the cells were suspended and incubated with 500 μL of 1 μM MitoTracker Red CMXRos (Beyotime Biotechnology, Shanghai, China) at 28 °C for 1 h in the dark, after which the cells were centrifuged, washed twice with PBS, and resuspended in 250 μL of PBS (Montoya et al., 2020). A total of 10 μL of cells was mounted onto a slide and observed with a Nikon Eclipse Ci-L microscope.

### 2.7 RNA-seq

#### 2.7.1 Sample preparation for transcriptomic analysis

*C. albicans* strains SC5314 and F0157 were first inoculated in SDB and grown at 28 °C with shaking at 180 rpm overnight. The cultures were then diluted to OD600 = 0.1 (Jenull et al., 2022). A total of 400 μL of inoculum was added to 40 ml of fresh SDB for 22 h of cultivation with an orbital shaker at 28 °C and 180 rpm. DMSO or drug was added at a final concentration of 1 μg/mL, and the mixture was shaken for another 2 h. After incubation with the drug, the cells were centrifuged at 3,900 rpm for 10 min and washed with fresh SDB three times. The total RNA of all the samples was extracted with an Ultrapure RNA Kit (CW0581M, CWBIO, Jiangsu, China) according to the manufacturer's instructions. The experiment was carried out in triplicate. The treatment group containing the drug-susceptible strain SC5314 was designated with “S,” correspondingly, the treatment group containing the drug-resistant strain F0157 was assigned as “R.” The 6 groups subjected to the corresponding treatments were labeled S_TPP-C_12_, S_TPP-C_14_, S_DMSO, R_ TPP-C_12_, R_ TPP-C_14_, and R_DMSO.

#### 2.7.2 RNA-seq profiling

RNA sequencing was performed by Shanghai Majorbio Biopharm Biotechnology Co., Ltd. (Shanghai, China) via an Illumina TruSeqTM RNA Sample Prep Kit according to the manufacturer's instructions. In brief, mRNAs were enriched, fragmented, and reverse transcribed to cDNA, which was followed by library construction and sequencing with the Illumina NovaSeq 6000 platform (pair-end, 2 × 150 bp read length).

#### 2.7.3 Transcriptomic data analysis

Filtering and quality control of the raw data were performed by fastp (https://github.com/OpenGene/fastp) with default parameters. HISAT2 (http://ccb.jhu.edu/software/hisat2/index.shtml) was used for alignment to the *C. albicans* SC5314 RefSeq genome (GCF_000182965.3). Assembly of the mapped reads was conducted with StringTie (https://ccb.jhu.edu/software/stringtie/) as described previously. The parameter “-cov_cutoff” was set to auto.

The gene and transcript expression levels were separately quantified using the expression quantification software RSEM v1.3.3 (http://deweylab.github.io/RSEM), and transcripts per million reads (TPM) was used as the quantification index.

Analysis of differential gene expression between samples/groups was performed using DESeq2 v1.24.0 software (http://bioconductor.org/packages/stats/bioc/DESeq2/). After the number of read counts of transcripts was obtained, the raw counts were analyzed with the default parameters, namely, Padjust < 0.05 and |log2FC| ≥ 1, to identify the differentially expressed genes (DEGs) between groups. Multiple-check calibration was performed via the Benjamini–Hochberg (BH) method. The functional classification annotation and functional enrichment of the DEGs were analyzed via the GO (Gene Ontology, http://www.geneontology.org/) and KEGG (Kyoto Encyclopedia of Genes and Genomes, http://www.genome.jp/kegg/) databases. Significant enrichment was considered when the corrected *P* value (Padjust) was < 0.05. Protein interaction network analysis for genes was performed via the STRING v11.5 database (http://string-db.org/), and network visualization was performed with networkX in Python. iPath 3.0 (http://pathways.embl.de) was utilized for visual analysis of metabolic pathway information on the basis of the DEGs of interest.

#### 2.7.4 qRT-PCR analysis of gene expression

qRT-PCR was performed using an ABI QuantStudio 6 flex (Thermo Fisher, USA). The same RNA as in the RNA-seq was used. One microgram of total RNA was reverse-transcribed to generate cDNA (Takara, Tokyo, Japan). The cDNA (20 ng/μL) was used as template, EvaGreen (Biotium, CA, USA) and qPCR Master Mix (Vazyme, Nanjing, China) were added according to the manufacturer's instructions. Genes that were mitochondrial-related and show significant up- or down-regulation were selected for validation; the specific genes and primer sequences were listed in the Supplementary Table S2. Relative quantitation method (Schmittgen and Livak, 2008) was employed to calculate transcription levels of selected genes, with actin serving as the reference gene.

### 2.8 Statistical analysis

The number of biological replicates is stated in each figure legend. The error bars represent the standard deviations (SDs) of the means. GraphPad Prism (version 10.3.1) was used for cytotoxicity analysis and qRT-PCR validation of TPP-C_12_ and TPP-C_14._ DESeq2 (Version 1.24.0) was used for differential gene expression analysis, and the multiple test correction method was the BH method. The method used for GO enrichment analysis was Fisher's exact test, and multiple test correction was performed by the BH method. When the corrected *P* value (Padjust) was < 0.05, the corresponding GO function or KEGG pathway was considered significantly enriched.

## 3 Results

### 3.1 Characterization of the *in vitro* antifungal activity of TPP-C_12_ and TPP-C_14_

TPP-C_12_ and TPP-C_14_ showed promising *in vitro* activity, with broad inhibitory effects against all the tested yeast and filamentous fungal species (Tables 1, 2). Specifically, the GM MIC ranges of TPP-C_12_ and TPP-C_14_ against yeast fungi were 1–6.4980 and 0.7071–1.6245 mg/L, respectively (Table 1, Figure 1B). TPP derivatives showed stable inhibitory activity toward all strains, including azole-resistant strains harboring ERG11 mutations (MICs as high as 32 mg/L for FLU, 2 mg/L for VOR, and 2 mg/L for ITA). In contrast, the MICs of the azoles varied widely, with POS ranging from 0.0156 to 2 mg/L, FLU from 0.0156 to 256 mg/L, VOR from 0.0156 to 8 mg/L, and ITA from 0.0313–4 mg/L. These results indicated that TPP-C_12_ and TPP-C_14_ are stable, effective and broad-spectrum antifungal agents with no species or strain specificity.

**Table 1:** MICs of TPP derivatives against yeast clinical isolates.

**Species**	**MIC index**	**MIC (mg/L)**					
		**TPP-C**_12_	**TPP-C**_14_	**FLU**	**VOR**	**ITA**	**PZ**
*Candida albicans*_resistant (8)	Range	1	1	8–32	0.5–2	0.125–2	0.125–0.5
	GM	1	1	19.0273	1	0.4585	0.3242
*Candida tropicalis* (10)	Range	1–2	1	0.5–64	0.0156–2	0.0313–0.5	0.0313–0.25
	GM	1.4142	1	2.1435	0.0769	0.1015	0.0948
*C. albicans* (10)	Range	1	0.5–1	0.5–64	0.0156–2	0.0313–2	0.0313–2
	GM	1	0.9330	4.2871	0.2499	0.1768	0.2177
*C. auris* (3)	Range	1–2	1	32–128	0.25–2	0.0625–0.25	0.0526–0.125
*C. krusei* (10)	Range	1	0.5–1	16–64	0.25–0.5	0.125–0.25	0.125–0.25
	GM	1	0.7071	25.9921	0.4353	0.1768	0.1649
***C. parapsilosis*** **complex**							
*C. parapsilosis* (10)	Range	2	1–2	0.0156–0.5	0.0156–0.0313	0.0313	0.0156–0.0625
	GM	2	1.6245	0.0272	0.0168	0.0313	0.0544
*C. orthopsilosis* (10)	Range	1	1	0.5–4	0.0156–0.0625	0.0313–0.25	0.0625–0.25
	GM	1	1	0.6156	0.0179	0.1088	0.125
*C. metapsilosis* (10)	Range	1–2	1–2	0.0625–2	0.0156–0.0625	0.0625–0.125	0.0625–0.125
	GM	1.0718	1.0718	1.1487	0.0292	0.0718	0.0718
***C. glabrata*** **complex**							
*C. glabrata* (10)	Range	4–8	1	8–256	0.25–8	0.125–2	0.25–2
	GM	5.6569	1	16	0.5359	0.4061	0.5359
*C. bracarensis* (3)	Range	4	1	2–8	0.0625–0.125	0.125–0.25	0.125–0.5
*C. nivariensis* (10)	Range	4–8	1	2–8	0.0313–0.25	0.0625–0.25	0.125–0.25
	GM	6.4980	1	3.4822	0.067	0.1350	0.1768
***C. haemulonii*** **complex**							
*C. haemolnii* (10)	Range	1	1	1–>256	0.0156–>8	0.0625–16	0.0156–0.5
	GM	1	1	78.7932	1.7408	0.3078	0.125
*C. duobushaemulonii* (10)	Range	1–2	1	4–32	0.0625–0.5	0.0625–0.25	0.0625–0.25
	GM	1.0718	1	6.9644	0.1436	0.1539	0.1015
*C. haemulonis var. vulnera* (4)	Range	1	1	0.5–64	0.0156–2	0.0313–0.25	0.0625–0.125
*Cryptococcus neoformans* (10)	Range	1	1	16	0.25	0.25	0.25
	GM	1	1	16	0.25	0.25	0.25
Total GM (128 isolates)	Range	0.5–8	0.5–2				
	GM	1.5174	1.0109				

The MIC is the concentration achieving 100% growth inhibition.

The GM refer to geometric mean MICs of strains within tested species.

FLU, fluconazole; VOR, voriconazole; ITA, itraconazole; PZ, posaconazole.

**Table 2:** MICs of TPP derivatives against clinical filamentous fungal isolates.

**Species**	**MIC index**	**MIC (mg/L)**					
		**TPP-C**_12_	**TPP-C**_14_	**FLU**	**VOR**	**ITA**	**PZ**
*Aspergillus flavus* (1)	Range	8	8	>256	2	2	0.5
*Aspergillus niger* (1)	Range	2	4	>256	2	2	0.5
*Aspergillus terreus* (1)	Range	4	4	>256	2	2	0.5
*Aspergillus nidulans* (1)	Range	4	4	>256	0.5	4	0.25
*Aspergillus fumigatus* (1)	Range	4	8	>256	4	2	1
*Trichophyton rubrum* (3)	Range	2	4	>256	2–8	2–4	2–4
*Trichophyton mentagrophytes* (2)	Range	4	4	>256	1–2	1–4	1–4
*Trichophyton soudanense* (1)	Range	2	2	>256	8	1	8
*Sporothrix globosa* (55)	Range	0.5–2	0.5–2	>256	16–32	16–32	>16
	GM	1.0134	1.0816	256	22.63	18.61	16

The MIC is the concentration achieving 100% growth inhibition.

The GM refer to geometric mean MICs of strains within tested species

FLU, fluconazole; VOR, voriconazole; ITA, itraconazole; PZ, posaconazole.

Interestingly, while TPP-C_12_ was less effective against the *C. glabrata* complex (GM concentrations: 4–6.4980 mg/L), TPP-C_14_ had no difference in inhibitory activity (GM: 1 mg/L) compared to other *Candida* species (GM: 0.7071–1.6245 mg/L). The MIC of TPP-C_14_ against all 128 strains was 1.0109 mg/L, lower than TPP-C_12_ (1.5173 mg/L). Although their structures differ by only two methyl groups, these results suggest TPP-C_12_ and TPP-C_14_ might have slightly different antifungal mechanisms, which require further analysis and verification. Overall, TPP-C_14_ emerged as a better candidate for wide antifungal use.

For *Aspergillus* species, the MIC ranges of TPP-C_12_ and TPP-C_14_ were 2–8 and 4–8 mg/L, respectively (Table 2). For the dermatophyte *Trichophyton* species, the MIC was 2–4 mg/L for both compounds. For the dimorphic *Sporothrix globosa* (mold phase, *n* = 55), the GM values for TPP-C_12_ and TPP-C_14_ were 1.0134 and 1.0816 mg/L, respectively. Compared to *Candida* species, the MIC values were higher for filamentous fungi, with both TPP-C_12_ and TPP-C_14_ having higher MIC values than conventional azoles. In addition, no synergistic interaction was observed against the tested strains (Supplementary Table S3).

### 3.2 Evaluation of *in vitro* cytotoxicity of TPP-C_12_ and TPP-C_14_

**Figure 2:**
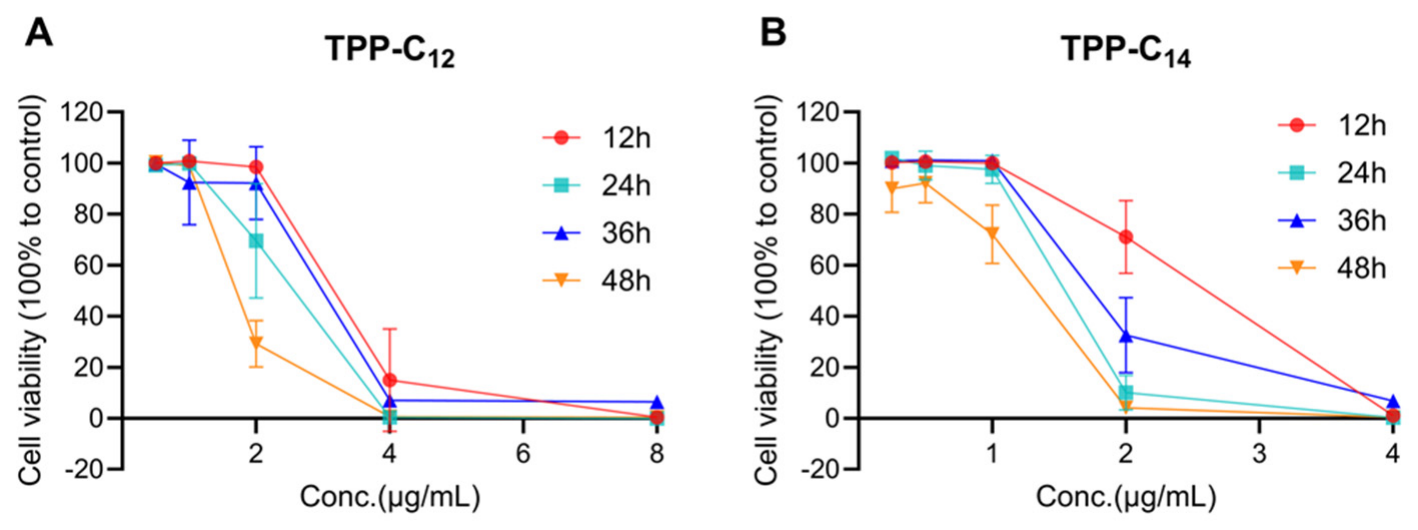
*In vitro* toxicity of TPP derivatives. Cell viability was assessed in AGS cell by CKK-8 following 12–48 h of treatment by TPP-C_12_ **(A)** and TPP-C_14_ **(B)**. Control cells were exposed to Dulbecco's Modified Eagle's Medium (DMEM). Mean values and SD from 5 biological replicates are plotted.

As shown in Figure 2, the cytotoxicity was dependent mainly on the drug concentration for both TPP-C_12_ and TPP-C_14_. In general, 2 mg/L was the cutoff for the *in vitro* cytotoxicity of both TPP-C_12_ and TPP-C_14_. When the drug concentration was 2 mg/L, the incubation time significantly affected the cytotoxicity of both drugs: cell viability decreased sharply from 98.46% to 29.22% for TPP-C_12_ and from 71.02% to 4.07% for TPP-C_14_ as the incubation time increased from 12 to 48 h. When the drug concentration was < 2 mg/L, the cells maintained high viability even when the incubation time increased to 48 h. When the drug concentration was >2 mg/L, both drugs exhibited extremely high cytotoxicity independent of the incubation time. In addition, TPP-C_14_ was more cytotoxic than TPP-C_12_ at the same concentration, especially at ≤ 2 mg/L, which was consistent with the antifungal susceptibility test results (where the MIC of TPP-C_14_ was lower than that of TPP-C_12_).

### 3.3 Transcriptomic profiles of *Candida albicans* under treatment with TPP-C_12_ and TPP-C_14_

**Figure 3:**
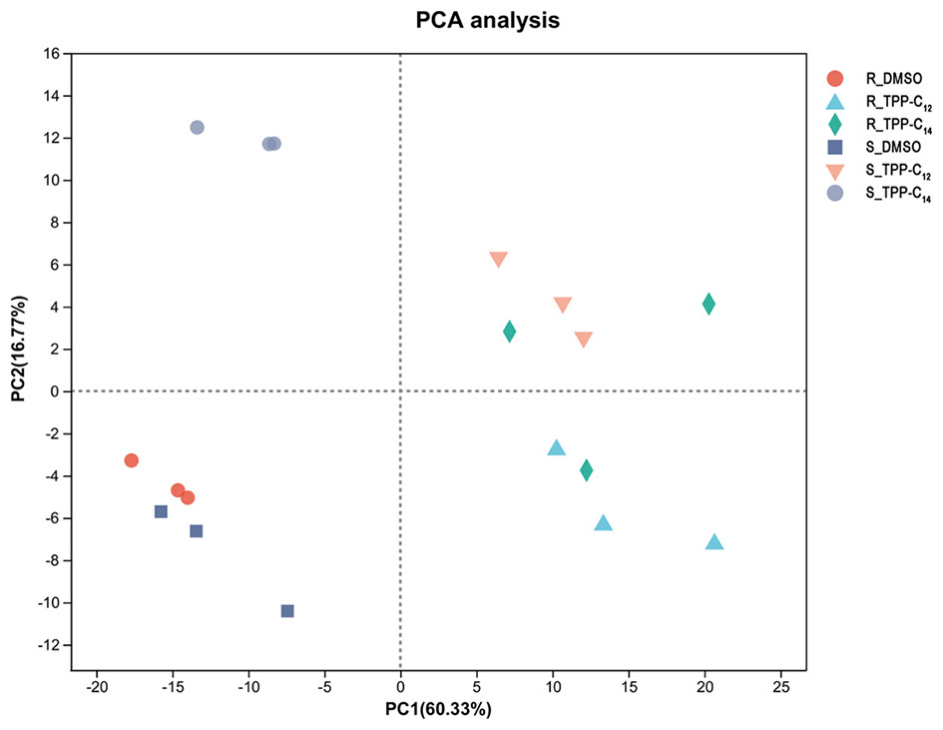
Principal component analysis (PCA) based on normalized RNA-seq read counts was used to evaluate the correlation and dispersion between different samples. The groups of dimethyl sulfoxide (DMSO)-treated and TPP derivative-treated *C. albicans* are shown in different colors.

Based on the sequenced data were of high quality (Supplementary Table S4), a total of 6,263 genes were annotated by aligning the assembled transcripts against six major databases. RSEM software was used to quantify the expression levels of the annotated genes, and TPM was used as a quantitative indicator (Supplementary Figure S1). Principal component analysis (PCA) revealed high quality of the RNA-seq data: the samples from the two control groups and four drug-treated groups were scattered (PC1, 60.33% variance), whereas the samples from the same group were aggregated (Figure 3). This was also confirmed by the correlation between samples (Supplementary Figure S2).

DEGs analysis revealed that, the S_TPP-C_14_ treatment contained 1,160 DEGs, which was only approximately 50% of the number of DEGs in the remaining three treatments (2209 DEGs for S_TPP-C_12_, 2,212 DEGs for R_TPP-C_12_, and 2,325 DEGs for R_TPP-C_14_; Table 3). In terms of sample correlation and PCA, the relationships between S_TPP-C_14_ and the control group were closer than those between S_TPP-C_14_ and the other three treatment groups, which was consistent with the lower number of DEGs in the S_TPP-C_14_ group.

**Table 3:** Differentially expressed genes in comparisons of TPP derivatives treated vs. control group.

**Diff_group**	**Up**	**Down**	**Total DEGs**
S_TPP-C_12__vs_S_DMSO	1,101	1,108	2,209
S_TPP-C_14__vs_S_DMSO	731	429	1,160
R_TPP-C_12__vs_R_DMSO	1,288	924	2,212
R_TPP-C_14__vs_R_DMSO	1,261	1,064	2,325
R_DMSO_vs_S_DMSO	276	214	490

### 3.4 DEGs analysis of *Candida albicans* in response to TPP-C_12_ and TPP-C_14_ treatment

**Figure 4:**
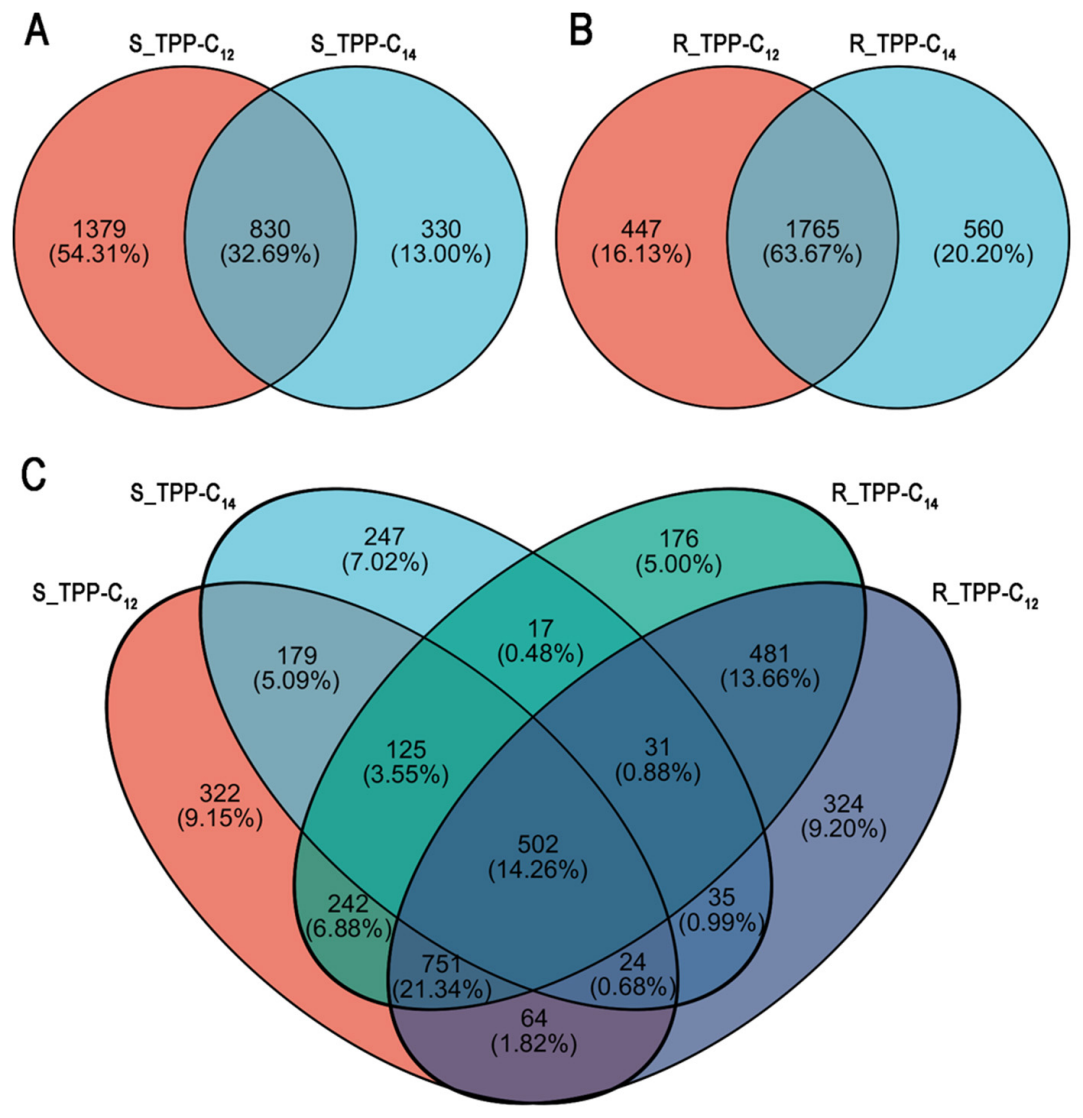
Venn diagram depicting the overlap of common genes differentially expressed (|log_2_-fold change| ≥ 1, Padjust < 0.05) between azoles-susceptible SC5314 and azoles-resistant F0157. Shared and unique DEGs identified in SC5314 **(A)** and F0157 **(B)** after treatment with TPP-C_12_ and TPP-C_14_. **(C)** Shared DEGs among all four treatments. The sum of the numbers inside each circle represents the total number of DEGs for that specific treatment, while the intersecting region of the circles indicates the number of DEGs shared between the treatments. For DEGs analysis, the multiple-testing correction method was BH (FDR correction with Benjamini–Hochberg).

A total of 830 shared DEGs were observed between the S_TPP-C_12_ and S_TPP-C_14_ groups (Figure 4A, Supplementary Figure S3). Among these shared DEGs, 567 genes were jointly upregulated, and 261 genes were jointly downregulated. Two genes (gene CAALFM-C206770WA; gene CAALFM-CR02560CA) were downregulated in the S_TPP-C_12_ group but upregulated in the S_TPP-C_14_ group. Notably, there was no significant difference in the inhibitory effects of TPP-C_12_ and TPP-C_14_ on azole-susceptible SC5314 (average MIC of 1 mg/L for both drugs), indicating that shared DEGs among both drug treatments were key genes responsible for the inhibition of *Candida* spp. For the azole-resistant F0157 strain, 2,212 and 2,325 genes were differentially expressed in the R_TPP-C_12_ and R_TPP-C_14_ groups, respectively (Table 3), among which 1,765 DEGs were shared by both groups. Among the shared DEGs, 1,058 were upregulated and 707 were downregulated (Figure 4B, Supplementary Figure S4).

Further analysis revealed 502 shared DEGs among all four treatments, including 401 upregulated DEGs and 98 downregulated DEGs (Figure 4C, Supplementary Figure S5). The remaining 3 shared DEGs were inconsistently upregulated/downregulated across the different groups. Considering that both drugs significantly inhibited the growth of *Candida* spp., the common DEGs among all four groups may be key genes for the antifungal activity of TPP-based derivatives.

### 3.5 Functional analysis of DEGs induced by TPP-C_12_ and TPP-C_14_ treatment

**Figure 5:**
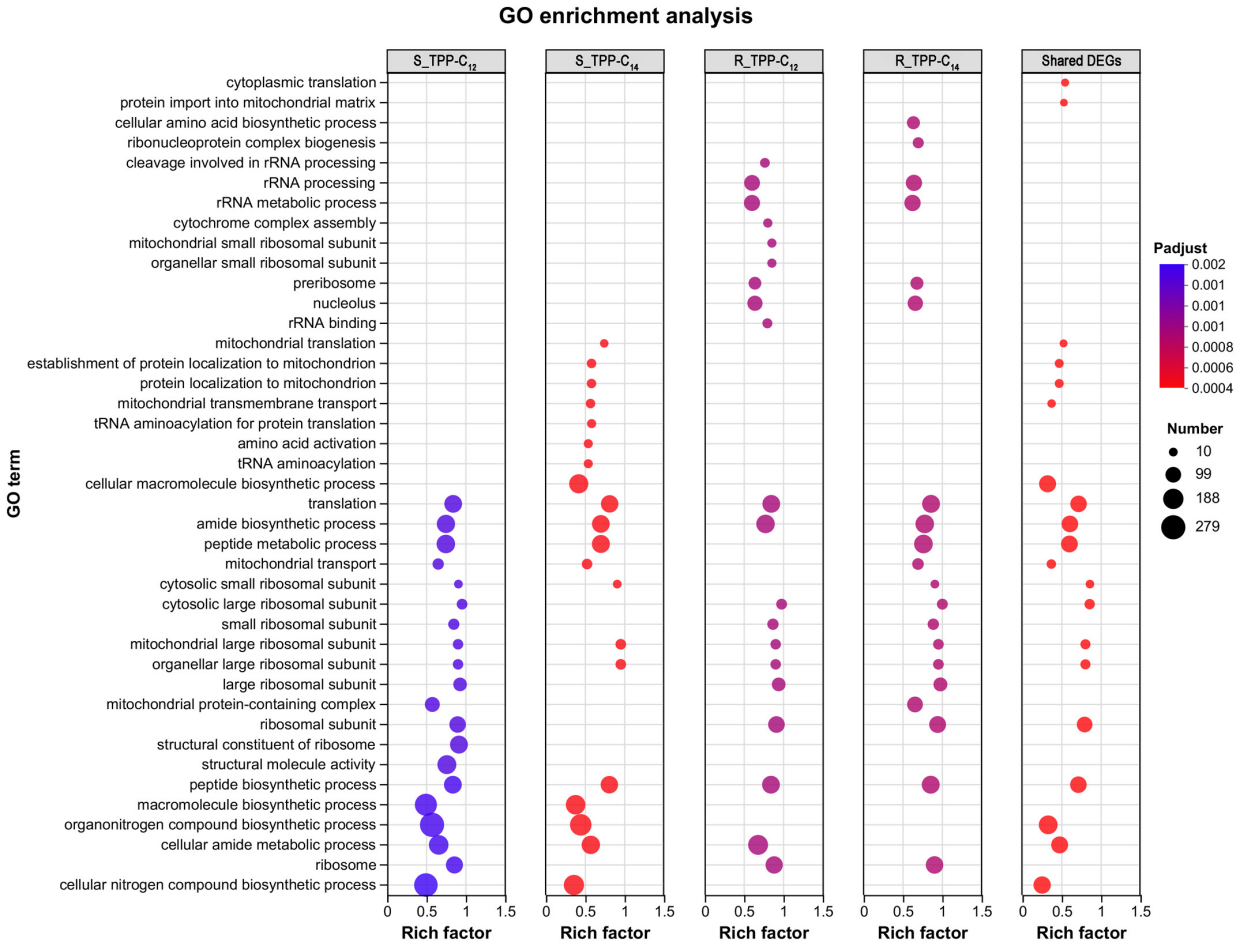
Bubble diagram of top 20 ranked GO terms of DEGs (|log_2_-fold change| ≥ 1, Padjust < 0.05). The vertical axis indicates GO terms and the horizontal axis represents the Rich factor. The enrichment degree was stronger with a bigger Rich factor. The size of dots indicates the number of genes in the GO term.

Gene Ontology (GO) enrichment analysis revealed the top 20 GO terms enriched by each individual treatment (Figure 5). For the azole-susceptible SC5314, both drugs led to the upregulation of genes related to translation and mitochondrial transport, including genes related to the cellular amide metabolic process, the cellular nitrogen compound biosynthetic process, the organonitrogen compound biosynthetic process, and the macromolecule biosynthetic process. The cellular component (CC) terms were associated mainly with the ribosome and mitochondrion (Figure 5, Supplementary Tables S5, S6, Supplementary Figures S6, S7). For the azole-resistant F0157, in addition to these upregulated genes, downregulation was observed in genes related to rRNA metabolic processes, the preribosome and nucleolus, indicating a broader involvement of the nucleus (Figure 5, Supplementary Tables S7, S8, Supplementary Figures S8, S9).

The shared DEGs among all 4 treatments were enriched mainly in mitochondrial transport, specifically in ribosome and mitochondrion (Supplementary Table S9, Supplementary Figure S10), indicating that the functions of the TPP derivatives were related to ribosome and mitochondrion, consistent with the chemical properties of TPP.

**Figure 6:**
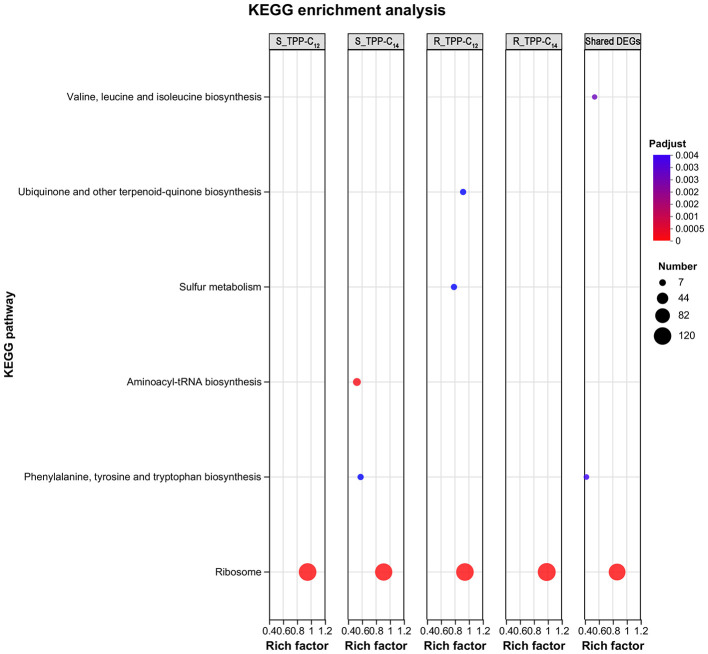
Bubble diagram of top 20 ranked KEGG pathways of DEGs (|log2-fold change| ≥ 1, Padjust < 0.05) for the indicated comparisons. The vertical axis represents the pathway name, and the horizontal axis represents the ratio of the number of enriched gene samples in this pathway to the number of background annotated genes. The size of the dots indicates the number of genes in the pathway, and the color of the dots corresponds to different Padjust ranges.

KEGG enrichment revealed that the ribosome was significantly enriched among all 4 treatment groups (Figure 6). Specifically, aminoacyl-tRNA biosynthesis and phenylalanine, tyrosine and tryptophan biosynthesis were enriched in the S_TPP-C_14_ group; while sulfur metabolism and ubiquinone and other terpenoid-quinone biosynthesis were enriched in the R_TPP-C_12_ group (Figure 6).

Analysis of shared DEGs among the 4 treatments focused on the ribosome, aminoacyl-tRNA biosynthesis, phenylalanine, tyrosine and tryptophan biosynthesis, and valine, leucine, and isoleucine biosynthesis, which were suggested as key mechanisms for the antifungal activity of TPP derivatives (Figure 6). Protein interaction network analysis revealed 12 key interacting protein nodes (Supplementary Figure S11), most of which were ribosomal proteins, indicating the importance of ribosomes in the drug action process. Further exploration of the “ribosome” pathway revealed 105 genes (Supplementary Table S10), including 28 mitochondrial ribosomal protein-encoding genes and 77 ribosomal protein-encoding genes, suggesting coordinated regulation of cytosolic ribosomes (CRs) and mitochondrial ribosomes (MRs).

Genes enriched in the aminoacyl-tRNA biosynthesis pathway encode aminoacyl-tRNA synthetases (ARSs) responsible for ligation of tRNAs with various amino acids (Figuccia et al., 2021), including glutamate, putative proline, phenylalanine, tyrosine, tryptophan, methionine, isoleucine, threonine, leucine, and aspartate (Supplementary Table S11), consistent with the annotation of the remaining two pathways (Supplementary Tables S12, S13).

### 3.6 Mitochondrial dysregulation mechanism of TPP derivatives

iPath 3.0 (http://pathways.embl.de) was used to visually analyze the metabolic pathways associated with the DEGs and illustrate the metabolic pathway information of the entire biological system after drug treatment (Supplementary Figure S12), revealing that the DEGs common to all 4 treatments were enriched in several pathways related to energy metabolism. In addition, compared with those in azole-susceptible SC5314, more pathways were involved in azole-resistant F0157 after treatment with both TPP-C_12_ and TPP-C_14_.

**Figure 7:**
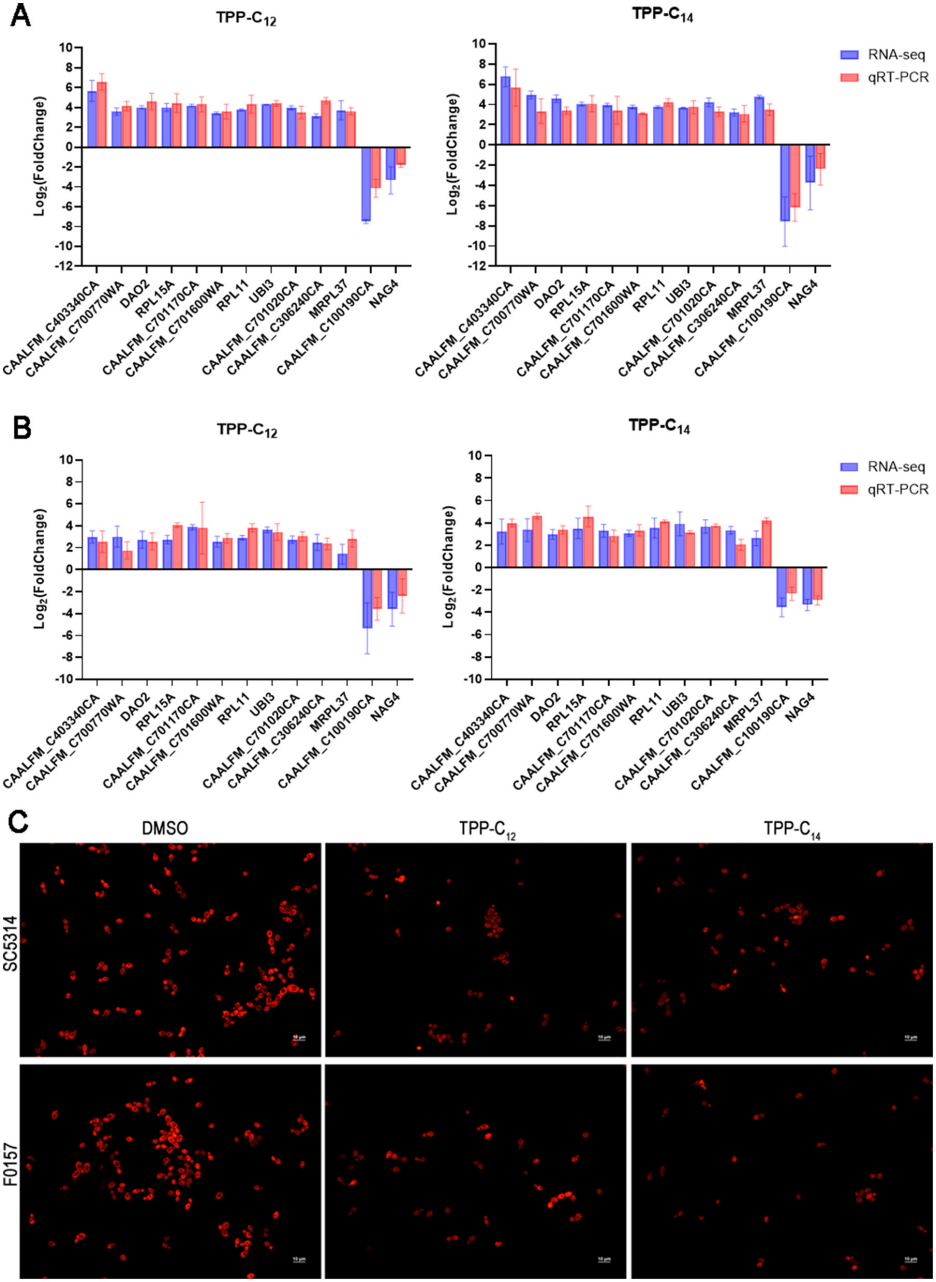
Validation of RNA-seq results. Expression levels of selected genes in the SC5314 **(A)** and F0157 **(B)** after treated with TPP-C_12_ and TPP-C_14_. X-axis represents gene names. Data represent the means ± SD from 3 biological replicates. Statistical significance of difference was determined by Mann-Whitney test. **(C)** Fluorescence microscopy observation of *C. albicans* mitochondria after treatment with TPP-C_12_ and TPP-C_14_. Azole-susceptible *C. albicans* SC5314C azole-resistant *C. albicans* F0157 were treated with TPP derivatives (final concentration of 1 μg/mL) or DMSO for 2 h. The cells were harvested, stained with MitoTracker Red CMXRos. The scale bar represents 10 μm.

To validate the RNA-seq results, we selected mitochondrial-related DEGs and those with large fold changes for qRT-PCR analysis. The qPCR data showed that the trend of gene-expression differences within both F0157 and SC5314 matched the RNA-seq findings, and no significant discrepancy was observed between the two methods, indicating the reliability of the transcriptomic data (Figures 7A, B).

To determine whether TPP derivatives affect mitochondrial function in pathogenic yeast, log-phase *C. albicans* (strains SC5314 and F0157) cells were exposed to TPP-C1_2_/TPP-C_14_ or DMSO and stained with MitoTracker Red CMXRos, which is dependent on the mitochondrial membrane potential for uptake. After incubation, both TPP-C_12_ and TPP-C_14_ treated cells showed decreased uptake of MitoTracker Red, and the fluorescence intensity was lower than that of the control group (Figure 7C). Drugs dissipate the mitochondrial proton motive force as part of their antifungal activity. No significant difference was observed between SC5314 and F0157, which indicated that the mitochondrial dysregulation mechanism of TPP derivatives was not associated with the intrinsic drug resistance of fungal cells.

## 4 Discussion

In recent decades, fungal infections have become an increasingly serious problem worldwide, posing a significant threat to public health, including the health of both immunocompromised and immunocompromised populations (Lee et al., 2021). According to global epidemiological analysis, *Candida, Aspergillus*, and *Cryptococcus* are the top three pathogenic fungal genera associated with invasive fungal infections (Robbins et al., 2017; Fisher et al., 2020; Lee et al., 2021; Strickland and Shi, 2021). Specifically, *C. albicans* is the primary pathogen for invasive candidiasis in hospitals. Research has revealed that there is a spectrum of *C. albicans* strains fully resistant to azole drugs, named Clade 1-α, and these have spread to a certain extent in China (Gong et al., 2023). The main nonalbicans species include *C. glabrata, C. tropicalis, C. parapsilosis*, and *C. krusei* (Pappas et al., 2018; Lass-Flörl and Steixner, 2023). Among them, *C. glabrata* is resistant to echinocandins, and *C. krusei* is intrinsically resistant to fluconazole (Pappas et al., 2018). A special drug-resistant clade of *C. tropicalis* (named AZR) have been revealed, where all strains in this clade are insensitive to fluconazole and voriconazole (Fan et al., 2023). In addition to the above species, new multidrug-resistant *Candida* species have emerged, such as *C. auris* discovered in the past decade and *C. haemulonii* in China (Chen et al., 2023). The two TPP derivatives reported in this study, TPP-C_12_ and TPP-C_14_, exhibited excellent antifungal effects against all the tested *Candida* species, including fluconazole-resistant strains.

Notably, there is a significant difference in the efficacy of the two drugs against *C. glabrata*. The target of TPP-derived drugs is mitochondria. According to previous reports, *C. glabrata* is tolerant to oxidative stress and may have mitochondrial defects (Ferrari et al., 2011; Usher et al., 2020; Brown et al., 2024), which may be responsible for the high MIC value of TPP-C_12_. While TPP-C_14_ is capable of overcoming the oxidative stress tolerance of *C. glabrata*, indicating differences in the molecular mechanisms of TPP-C_12_ and TPP-C_14_, and further research is needed to clarify the specific pathways involved.

As another important class of yeast phase fungi, the genus *Cryptococcus* can be divided into two complex types: *C. neoformans* and *C. gattii* (Ortiz and Hull, 2024). *C. neoformans* is listed as a critical priority in the World Health Organization (WHO) priority list of pathogenic fungi. As to filamentous fungi, *Aspergillus* is the most common pathogen, and causes 2.11 million invasive aspergillosis cases annually with an estimated annual mortality rate of 1.8 million (85.2%; Denning, 2024). *A. fumigatus* accounts for 90% of aspergillosis infections, while *A. flavus, A. nidulans, A. terreus*, and *A. niger* are also common pathogens in clinic (Pérez-Cantero et al., 2020; Lass-Flörl and Steixner, 2023). In addition to invasive infections, superficial fungal infections, primarily caused by dermatophytes, affect 25% of the global population and impose a significant disease burden. The *Trichophyton rubrum* complex is the dominant dermatophytes worldwide. In addition, the increasing prevalence of *T. mentagrophytes*, is becoming an serious problem worldwide, particularly terbinafine-resistant infections associated with *T. indotineae* (Su et al., 2019; Cornet et al., 2021; Shao et al., 2025). In this study, both TPP-C_12_ and TPP-C_14_ showed strong antifungal effects against all the pathogenic fungi mentioned, providing new options for the clinical treatment of fungal infections.

Owing to its ability to target and accumulate in mitochondria, TPP is widely used in the design of mitochondrion-targeted compounds, including anticancer, antiparasitic, antioxidant, and antifungal agents. TPP-based novel antifungal compounds have been developed through linkage with azole molecules, gallic acid and chemical groups (Wang et al., 2021; Zhang et al., 2023; Valderrama et al., 2024). Valderrama et al. linked gallic acid to TPP via C10 and C12 linkers to obtain TPP^+^-C_10_ and TPP^+^-C_12_, which exhibit strong inhibitory effects on the growth of *C. albicans*. The MIC values differ by two-fold between drug-resistant and drug-sensitive strains. The two TPP compounds we report in this study had similar effects on sensitive and resistant *Candida* strains, with MICs of 1 μg/mL, lower than those reported by Victoria. Notably, in Victoria's study, the MIC was defined as the concentration that led to 50% inhibition of fungal growth, while in this study, the MIC was defined as 100% inhibition, indicating that TPP-C_12_ and TPP-C_14_ have stronger antifungal effects.

Wang's research showed that the length of the alkyl linkers clearly affects the activity. When the length of the carbon chain was increased to more than eight carbons, the activity improved significantly, which was consistent with our results: TPP-C_14_ with a C_14_ chain had a better antifungal effect than TPP-C_12_ with a C_12_ chain. In Valderrama's study, TPP^+^-C_10_ and TPP^+^-C_12_ inhibited oxygen consumption and reduced the mitochondrial membrane potential and ATP production, indicating that these compounds can cause mitochondrial dysfunction. In this study, both compounds reduce the absorption of MitoTracker Red. Analysis of the molecular mechanisms revealed that these two compounds act mainly on mitochondria. The GO enrichment and KEGG enrichment of DEGs were directed toward mitochondria and ribosomes, which was consistent with the above results.

When the concentration is 2 mg/L, corresponding to approximately 3.91 μM for TPP-C_12_ and 3.71 μM for TPP-C_14_, the cytotoxicity of two compounds is relatively low. Above this concentration, both compounds exhibit markedly higher cytotoxicity. According to the previous research, the cytotoxicity of TPP-derived compounds can vary widely. Martins et al. (2024), presents the cytotoxicity of dioxidovanadium(V) complexes (C1–C5) with a TPP moiety, concentrations of C2, C4, and C5 at 0.016, 0.0103, and 0.0122 mM, respectively, had virtually no effect on the proliferation of HaCaT cells, showing no significant difference compared with the control group. Conversely, in the work of Michał Sulik, a series of salinomycin and monensin are conjugated with TPP^+^, synthesized derivatives are labeled as 1a−1f and 2a−2f (Sulik et al., 2025). Compound 1f display IC50 values of 0.3–1.7 μM across eight tested cell lines, including six human cancer cell lines (SW480, SW620, PC3, MDA-MB-231, A549, and MiaPaCa) and two non-malignant cell lines (HaCaT and V79), indicating high cytotoxicity; whereas compound 2e has IC50 values ranging from 28.1 to >100 μM, suggesting relatively low cytotoxicity. It is also noteworthy that, in the present study, the MIC values for filamentous fungi *Aspergillus* spp. and *Trichophyton* spp. (a total of 10 isolates) exceed 2 mg/L, whereas the MICs for the yeast-phase pathogens *Candida* spp. and *Cryptococcus* spp. (128 isolates) and the dimorphic *Sporothrix globosa* (55 isolates) are generally ≤ 2 mg/L. Therefore, future applications could focus primarily on infections caused by yeast-phase fungi such as *Candida* and *Cryptococcus*. Moreover, a more comprehensive toxicity assessment is required; subsequent research should incorporate additional cell lines and *in-vivo* experiments to further refine the safety profile of TPP-C_12_ and TPP-C_14_.

Based on the results of this study, DEGs encoding mitochondrial ribosomal proteins (MRPs, including 37S, 54S), ribosomal proteins (also known as cytotoxic ribosomal proteins; CRPs, including 40S, 60S), and aminoacyl tRNA biosynthesis proteins (including a series of amino acid tRNA ligases) were identified. To clarify the associations and potential mechanisms of these DEGs, it is essential to understand the process of mitochondrial biogenesis in eukaryotic cells.

In most eukaryotic cells, the normal functioning of mitochondria depends on the expression of the mitochondrial genome and nuclear genes (Morgenstern et al., 2017). In short, Mitochondrial ribosomal proteins (MRPs) are encoded in the nuclear genome, synthesized on cytoplasmic ribosomes, and then introduced into the mitochondrial matrix, where they are incorporated into preribosome complexes (Kummer and Ban, 2021). During mitochondrial translation, aminoacyl-tRNA synthetases (ARS) ensures the correct connection between each amino acid and its homologous tRNA (Ibba and Soll, 2000), which is encoded by nuclear genes and then translated into mitochondria in the cytoplasm. Balancing the production of mitochondrial and cytoplasmic proteins is crucial for establishing respiratory chain complexes. Moreover, in eukaryotic cells, the cytoplasmic 80S ribosome is composed of two subunits: the 40S small subunit and the 60S large subunit (Dörner et al., 2023). The 74S mitochondrial ribosome in yeast is composed of a 37S subunit and a 54S subunit (Greber and Ban, 2016).

**Figure 8:**
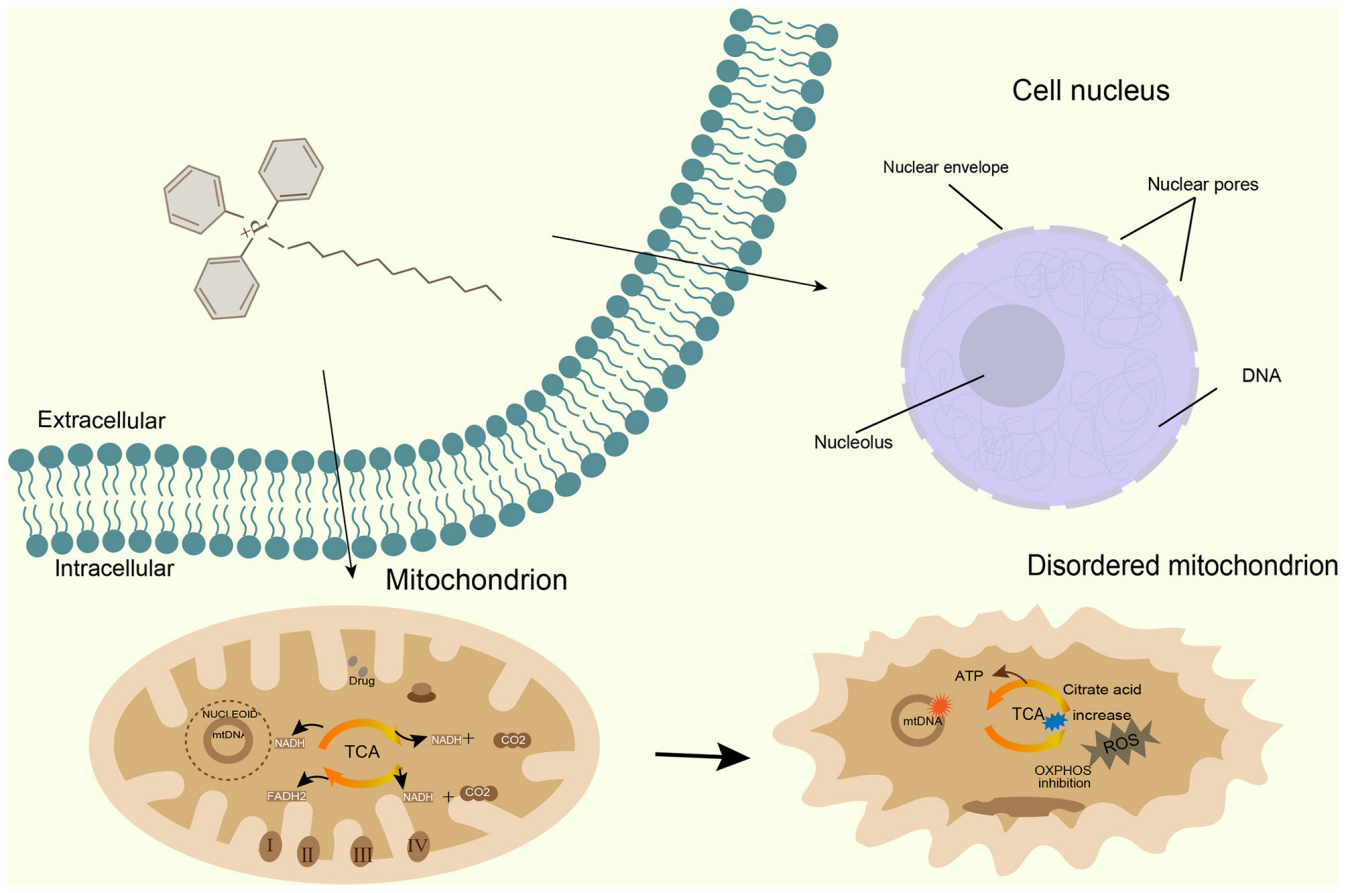
A schematic diagram illustrating antifungal effects of TPP derivatives on *C. albicans*.

According to the process of mitochondrial development and the results of this study, we propose a molecular mechanism of action for the compound (Figure 8): after drug molecules enter the cell, they enter the nucleus and affect the nuclear DNA encoding ARSs and MRPs. Moreover, by regulating cytoplasmic ribosomal protein-encoding genes, they affect protein translation and inhibit the synthesis of MRPs necessary for mitochondrial function. On the other hand, mitochondrial accumulation prevents the normal synthesis of OXPHOS complexes encoded by mtDNA and affects the mitochondrial membrane potential, leading to mitochondrial disruption, ultimately resulting in cell death.

## 5 Conclusion

Here, we have reported that two derivatives of the mitochondrion-targeted compound triphenylphosphonium (TPP), TPP-C_12_ and TPP-C_14_, exhibit broad-spectrum antifungal activity against pathogenic yeasts and molds, including resistant clinical isolates. *In vitro* toxicity profiles indicate that they are relatively safe for human cells at fungal MICs. RNA-seq revealed that the derivatives interfere with mitochondrial and ribosomal functions by coregulating mitochondrial and nuclear genes, leading to mitochondrial dysfunction and cell death. Taken together, TPP-C_12_ and TPP-C_14_ are stable, effective and broad-spectrum antifungal agents with no species or strain specificity. This study provides a broad scope for optimization and development of the antifungal activity of this class of molecules.

## Data Availability

The data presented in the study are deposited in the NCBI repository, BioProject Accession No: PRJNA1311176.
