# Supplementary material for: Broad-spectrum antifungal activity of C12/C14 alkyl triphenylphosphonium salts (TPP-C12 and TPP-C14) against clinically relevant pathogens
Source: Front Microbiol. 2026 Jan 26;16:1693528. doi: 10.3389/fmicb.2025.1693528 (PMC12883766; doi:10.3389/fmicb.2025.1693528)
Supplement: Supplementary file 7 [file Data_Sheet_1.docx]

Supplementary Material

## Supplementary Figures


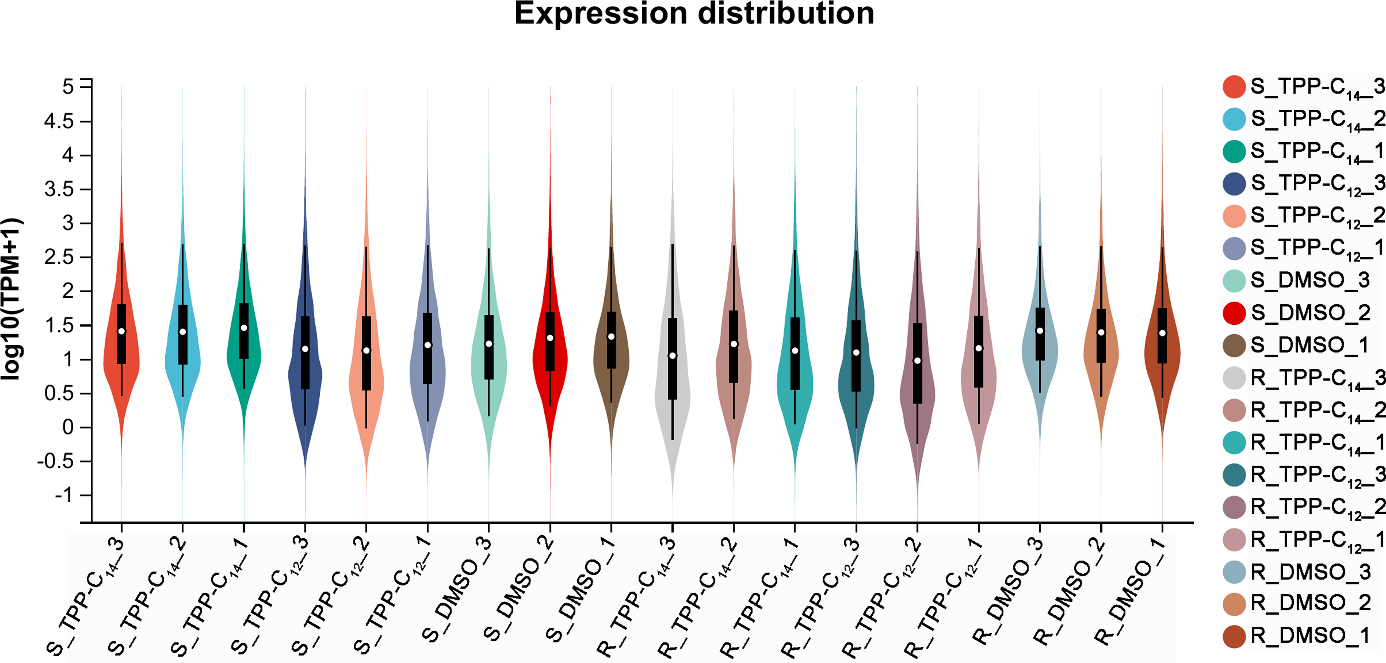


**Supplementary Figure 1.** The overall distribution of transcript expressions of samples. The X-axis indicates the sample names, while the Y-axis displays log10-transformed expression values (after adding 1). Each color corresponds to an individual sample. The expanded box region highlights the area where gene expression levels are most densely concentrated across all samples.


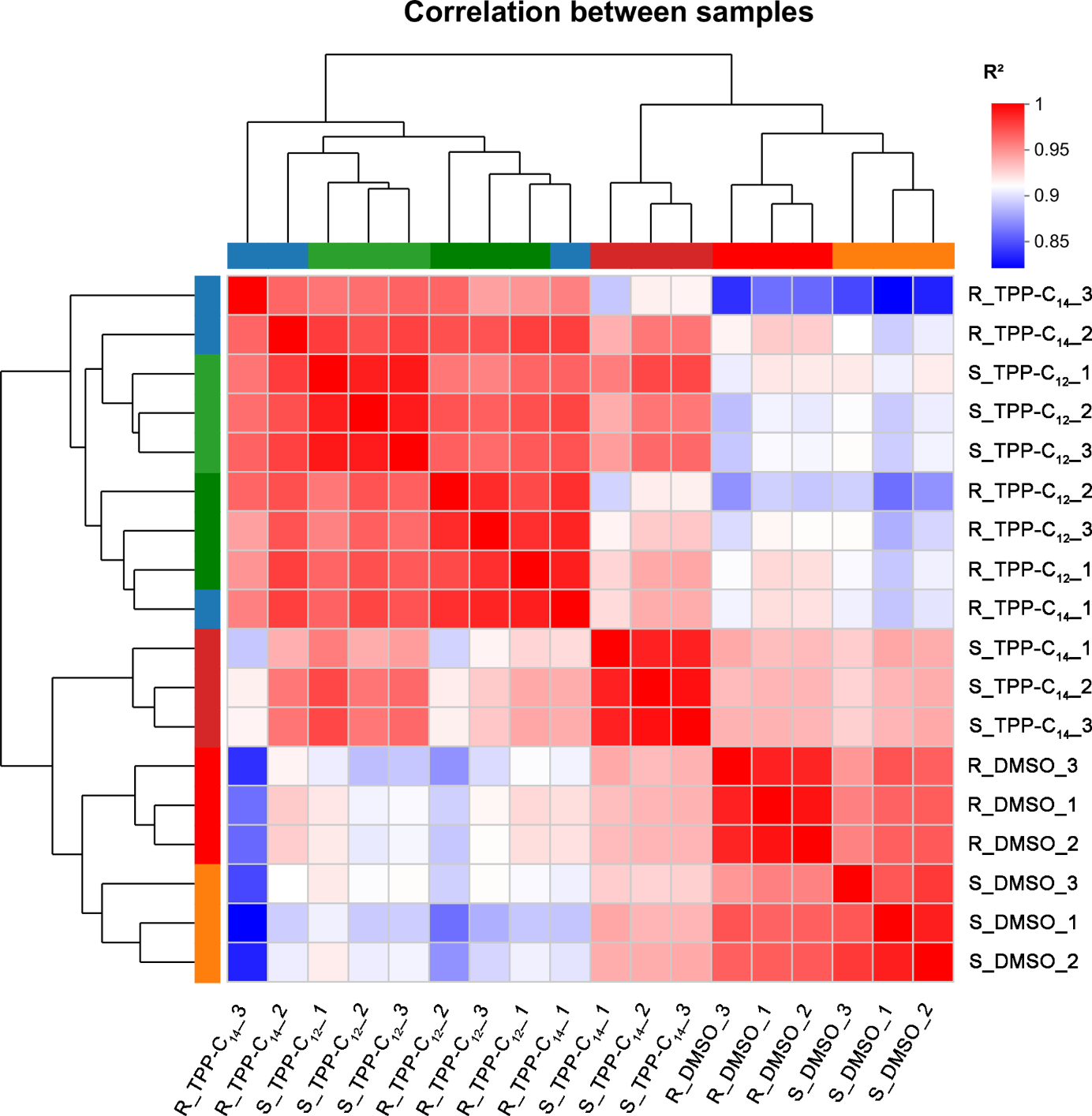


**Supplementary Figure 2.** Correlation analysis of the samples for differential expression analysis. The right and bottom sides show sample names; the left and top sides show sample clustering. Different colored squares represent the correlation levels between two samples.


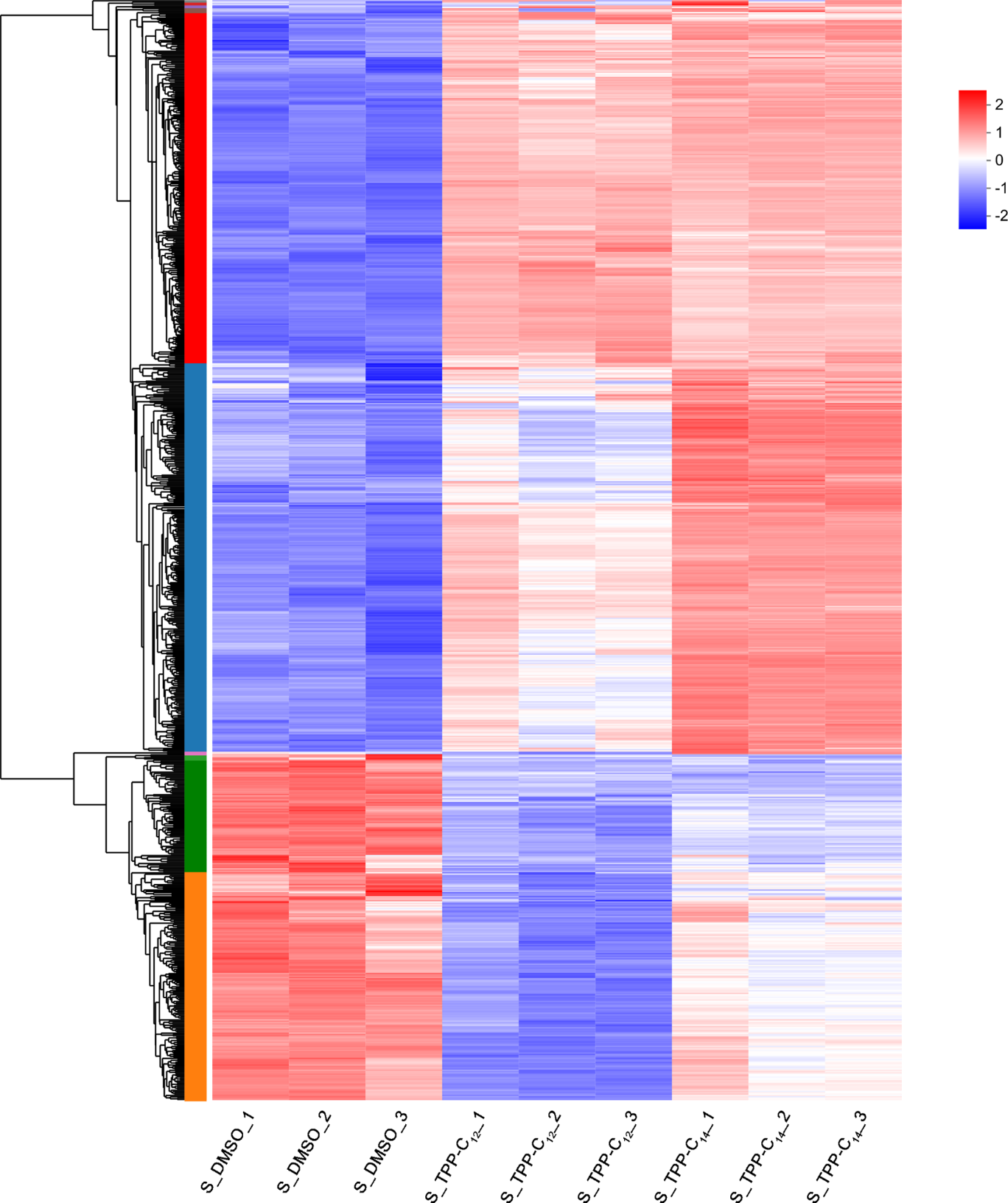


**Supplementary Figure 3.** Cluster analysis of 830 shared DEGs in azole-susceptible SC5314 following TPP-C12 and TPP-C14 Treatments. Columns represent individual samples, while rows correspond to genes. Color intensity reflects Z-score normalized expression levels (red: upregulated; blue: downregulated). Left panel: Gene clustering dendrogram and subcluster module map. Right panel: Gene names. Proximity between gene branches indicates similarity in expression patterns. Upper panel: Sample clustering dendrogram. Lower panel: Sample labels. Closer sample branches denote higher concordance in gene expression profiles.
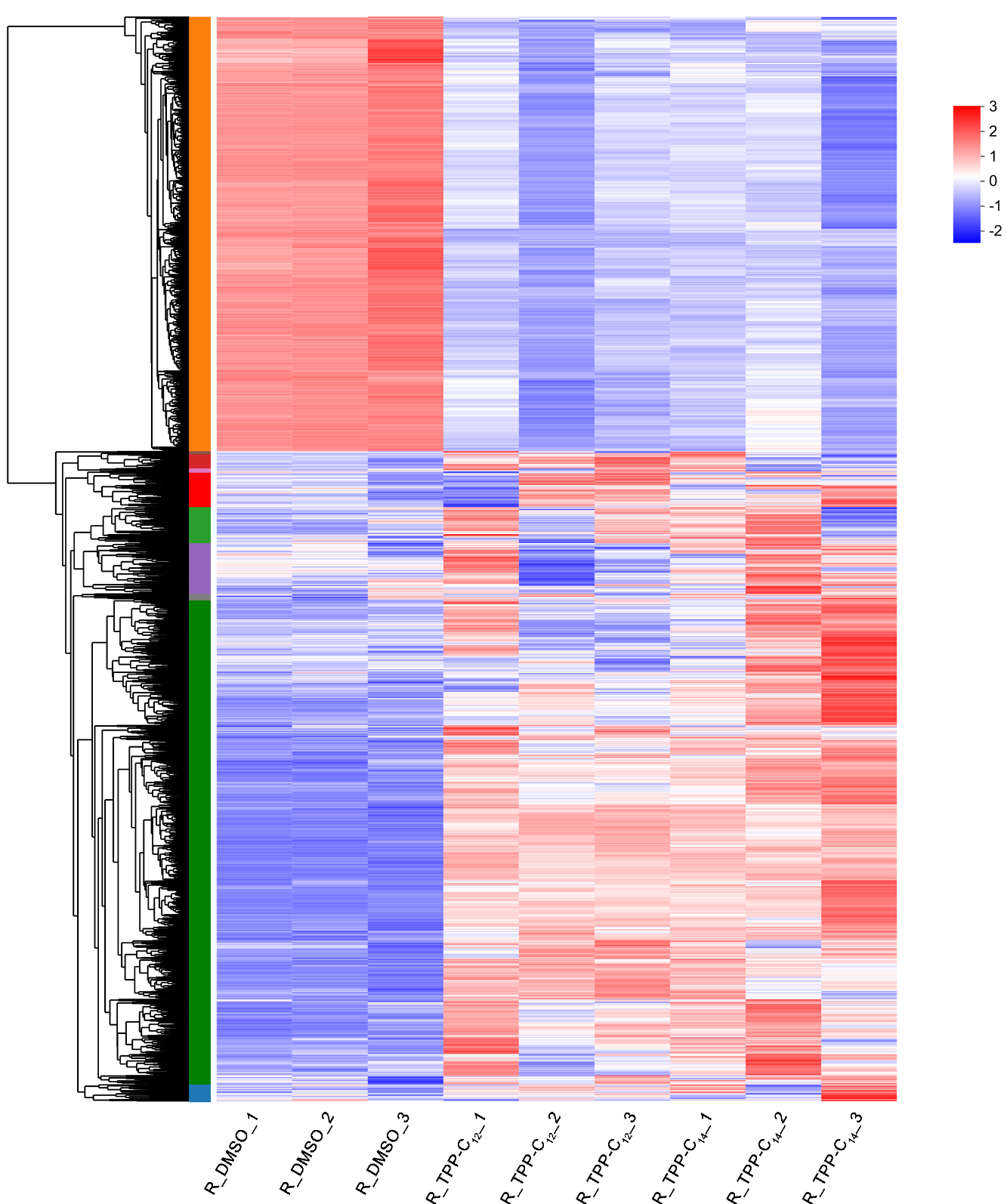


**Supplementary Figure 4.** Cluster analysis of 1765 shared DEGs in azole-resistant F0157 following TPP-C_12_ and TPP-C_14_ Treatments. Columns represent individual samples, while rows correspond to genes. Color intensity reflects Z-score normalized expression levels (red: upregulated; blue: downregulated). Left panel: Gene clustering dendrogram and subcluster module map. Right panel: Gene names. Proximity between gene branches indicates similarity in expression patterns. Upper panel: Sample clustering dendrogram. Lower panel: Sample labels. Closer sample branches denote higher concordance in gene expression profiles.


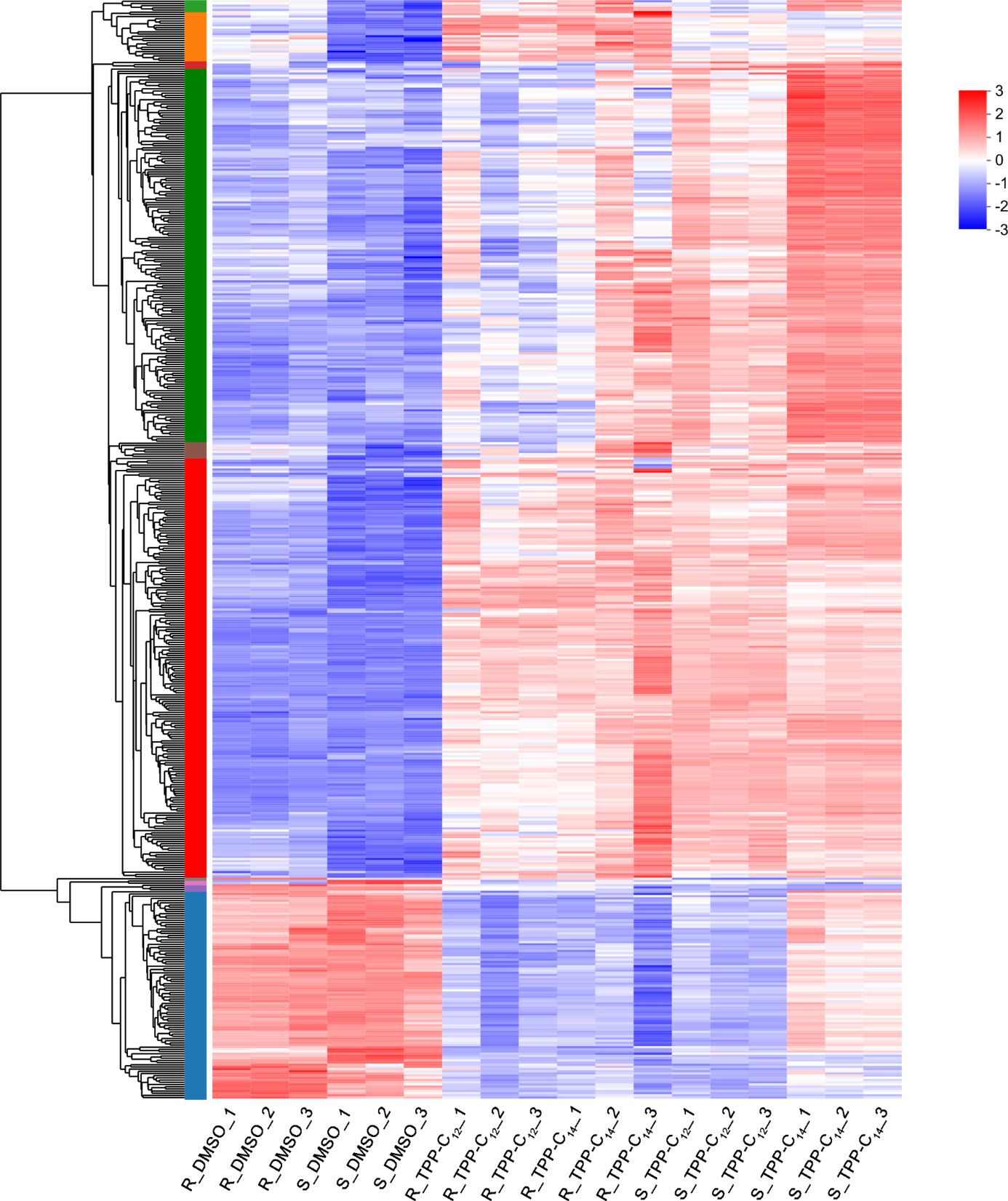


**Supplementary Figure 5.** Cluster analysis of 502 shared DEGs among 4 treatment groups. Columns represent individual samples, while rows correspond to genes. Color intensity reflects Z-score normalized expression levels (red: upregulated; blue: downregulated). Left panel: Gene clustering dendrogram and subcluster module map. Right panel: Gene names. Proximity between gene branches indicates similarity in expression patterns. Upper panel: Sample clustering dendrogram. Lower panel: Sample labels. Closer sample branches denote higher concordance in gene expression profiles.


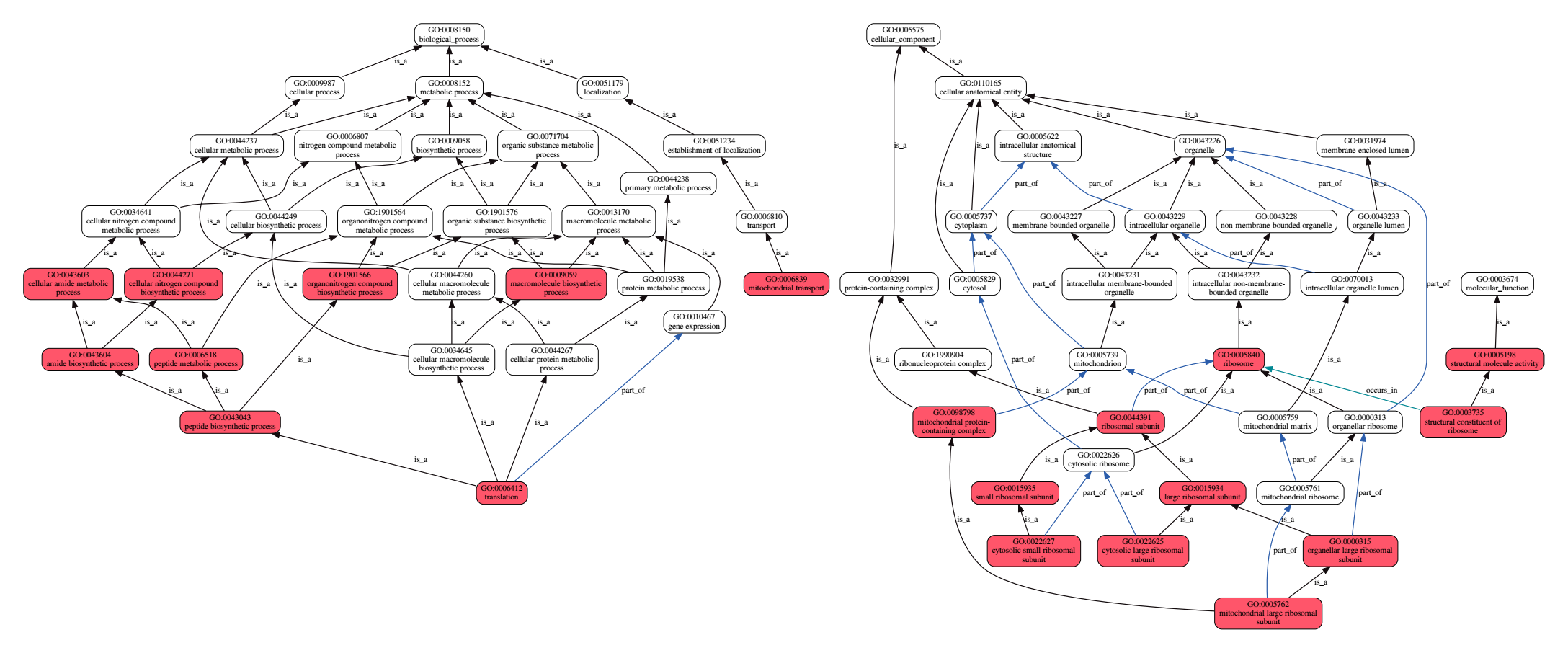


**Supplementary Figure 6.** Directed acyclic graph of GO enrichment of DEGs in azole-susceptible SC5314 following TPP-C_12_ treatment. The Gene Ontology (GO) analysis comprises three categories: Biological Process, Cellular Component, and Molecular Function, where each box represents a GO term, with color-coded boxes indicating significantly enriched terms (intensity of red corresponds to enrichment significance); directed edges in the directed acyclic graph (DAG) denote hierarchical relationships between GO terms using colored arrows: black arrows for "is_a" (indicating subset relationships, e.g., A is_a B implies A is a subclass of B), blue arrows for "part_of" (e.g., nucleus part_of cell, where the presence of C necessitates its containment within D).


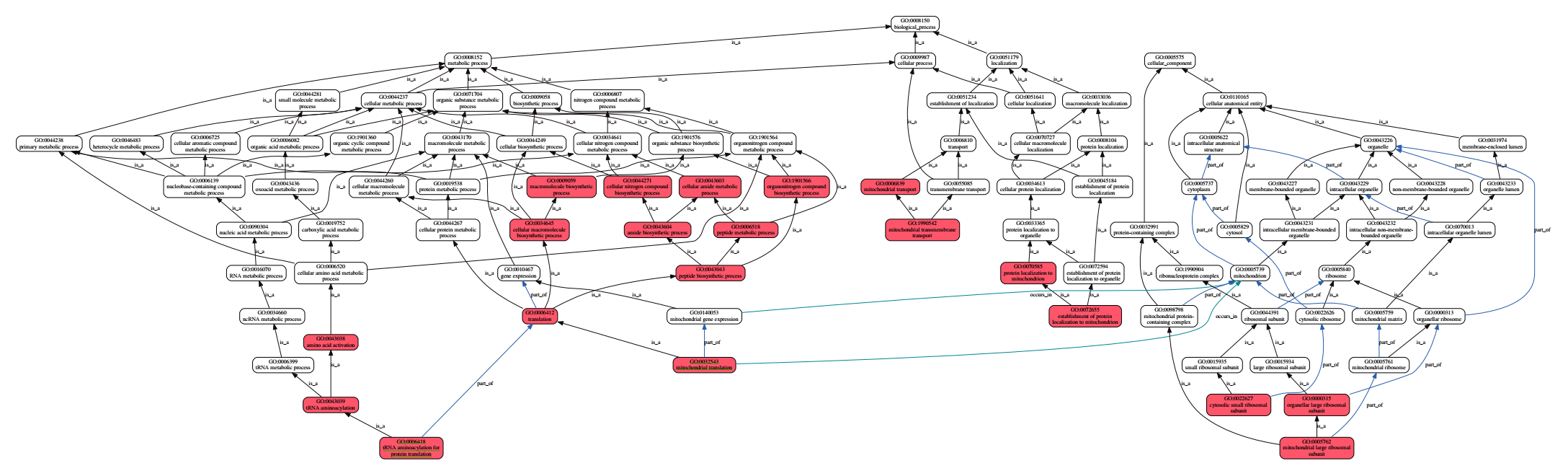


**Supplementary Figure 7.** Directed acyclic graph of GO enrichment of DEGs in azole-susceptible SC5314 following TPP-C_14_ treatment. The Gene Ontology (GO) analysis comprises three categories: Biological Process, Cellular Component, and Molecular Function, where each box represents a GO term, with color-coded boxes indicating significantly enriched terms (intensity of red corresponds to enrichment significance); directed edges in the directed acyclic graph (DAG) denote hierarchical relationships between GO terms using colored arrows: black arrows for "is_a" (indicating subset relationships, e.g., A is_a B implies A is a subclass of B), blue arrows for "part_of" (e.g., nucleus part_of cell, where the presence of C necessitates its containment within D).


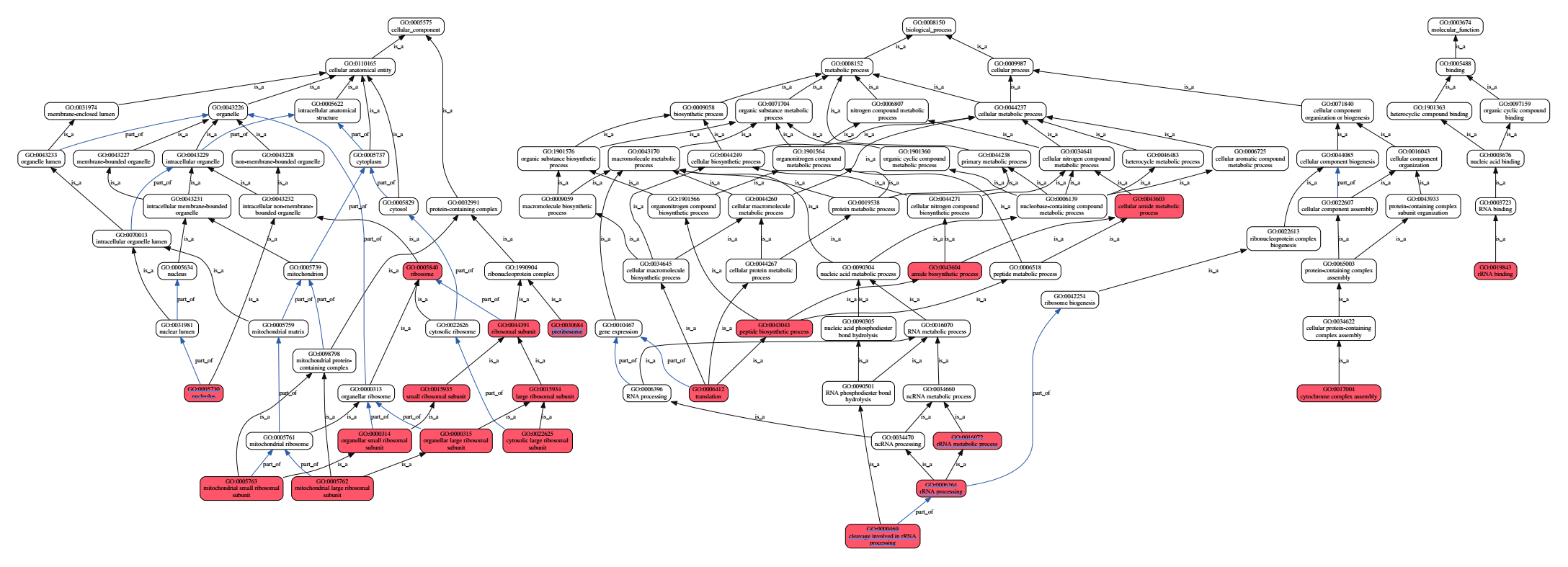


**Supplementary Figure 8.** Directed acyclic graph of GO enrichment of DEGs in azole-resistant F0157 following TPP-C_12_ treatment. The Gene Ontology (GO) analysis comprises three categories: Biological Process, Cellular Component, and Molecular Function, where each box represents a GO term, with color-coded boxes indicating significantly enriched terms (intensity of red corresponds to enrichment significance); directed edges in the directed acyclic graph (DAG) denote hierarchical relationships between GO terms using colored arrows: black arrows for "is_a" (indicating subset relationships, e.g., A is_a B implies A is a subclass of B), blue arrows for "part_of" (e.g., nucleus part_of cell, where the presence of C necessitates its containment within D).


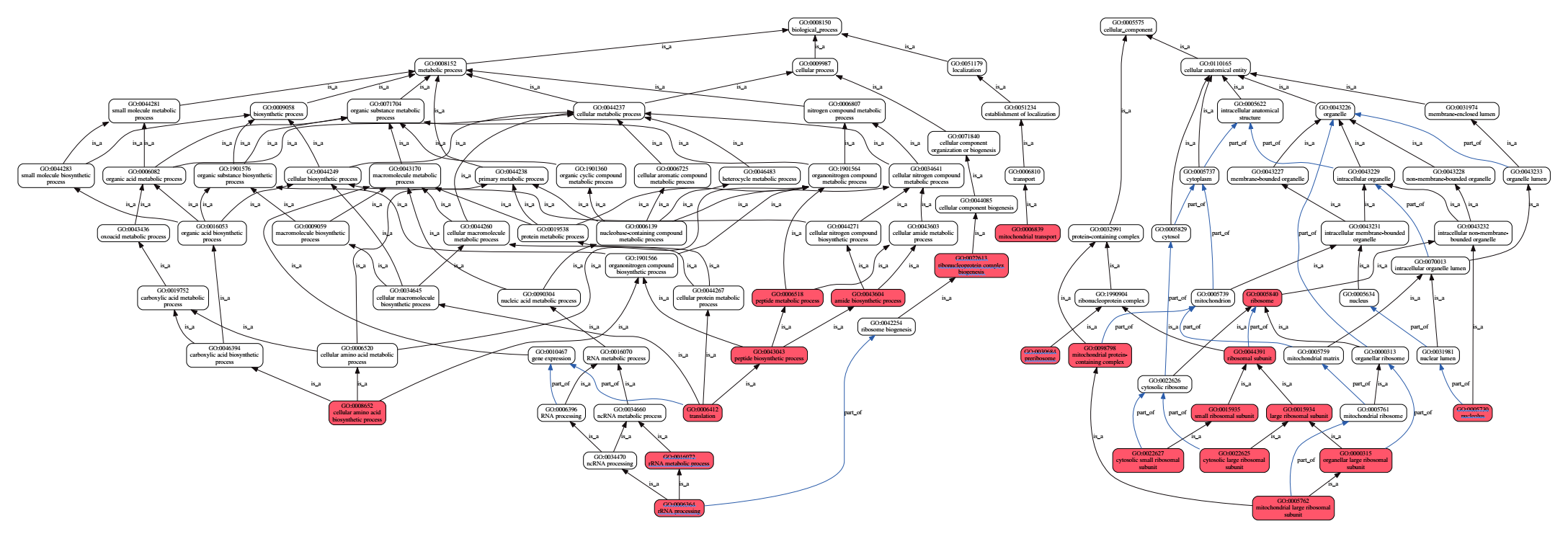


**Supplementary Figure 9.** Directed acyclic graph of GO enrichment of DEGs in azole-resistant F0157 following TPP-C_14_ treatment. The Gene Ontology (GO) analysis comprises three categories: Biological Process, Cellular Component, and Molecular Function, where each box represents a GO term, with color-coded boxes indicating significantly enriched terms (intensity of red corresponds to enrichment significance); directed edges in the directed acyclic graph (DAG) denote hierarchical relationships between GO terms using colored arrows: black arrows for "is_a" (indicating subset relationships, e.g., A is_a B implies A is a subclass of B), blue arrows for "part_of" (e.g., nucleus part_of cell, where the presence of C necessitates its containment within D).


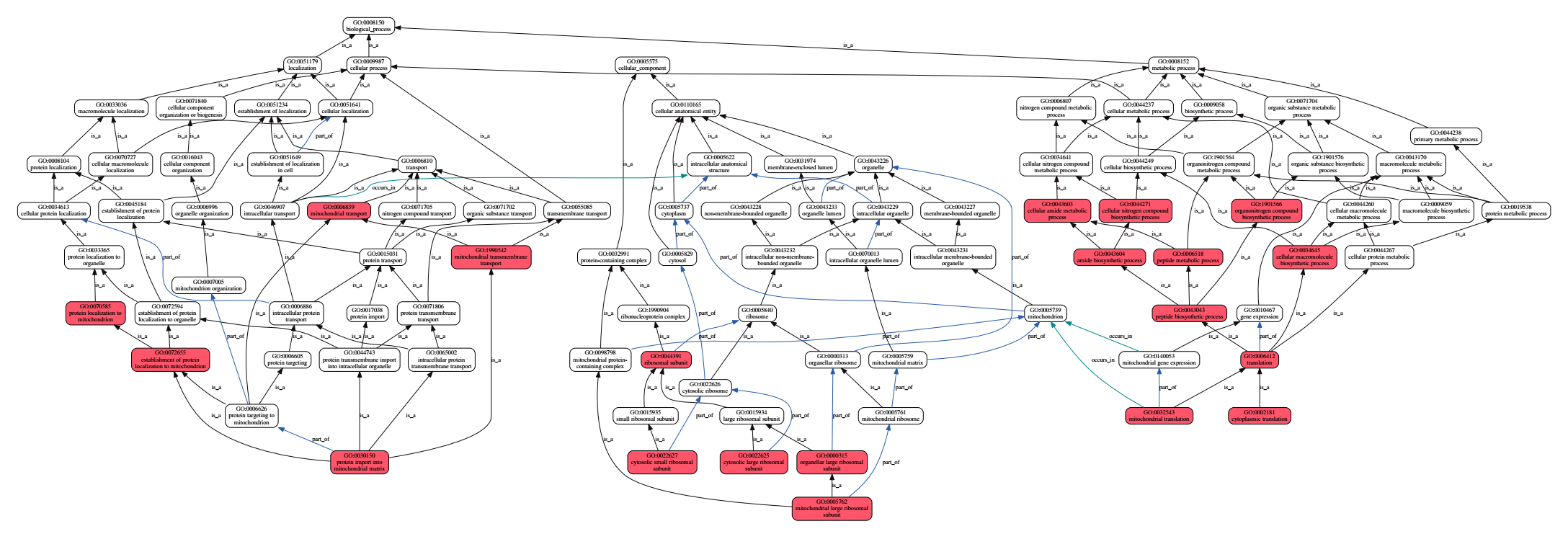


**Supplementary Figure 10.** Directed Directed acyclic graph of GO enrichment of shared DEGs among 4 treatment groups. The Gene Ontology (GO) analysis comprises three categories: Biological Process, Cellular Component, and Molecular Function, where each box represents a GO term, with color-coded boxes indicating significantly enriched terms (intensity of red corresponds to enrichment significance); directed edges in the directed acyclic graph (DAG) denote hierarchical relationships between GO terms using colored arrows: black arrows for "is_a" (indicating subset relationships, e.g., A is_a B implies A is a subclass of B), blue arrows for "part_of" (e.g., nucleus part_of cell, where the presence of C necessitates its containment within D).


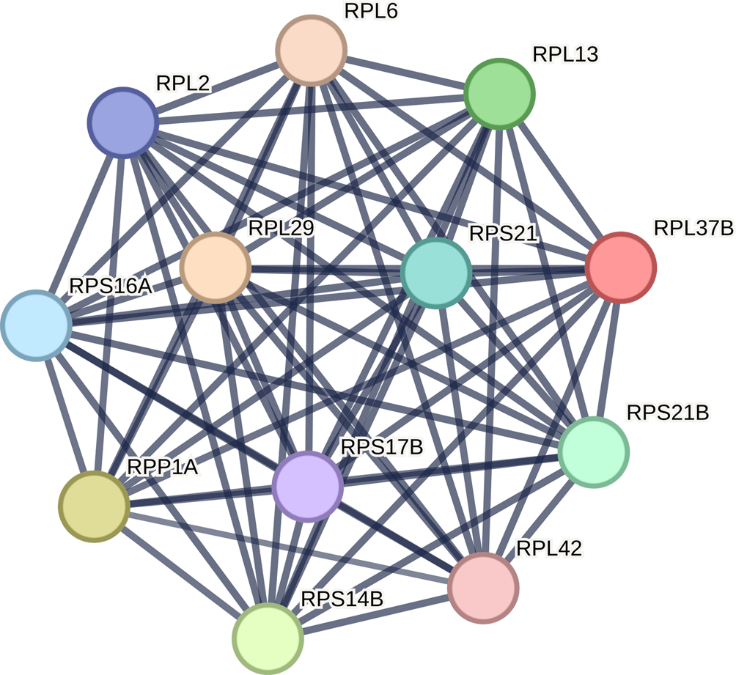


**Supplementary Figure 11.** Interaction analysis of common DEGs among 4 treatments. The analysis was generated with STRING database (<http://string-db.org/>), based on a PPI network of Candida albicans.


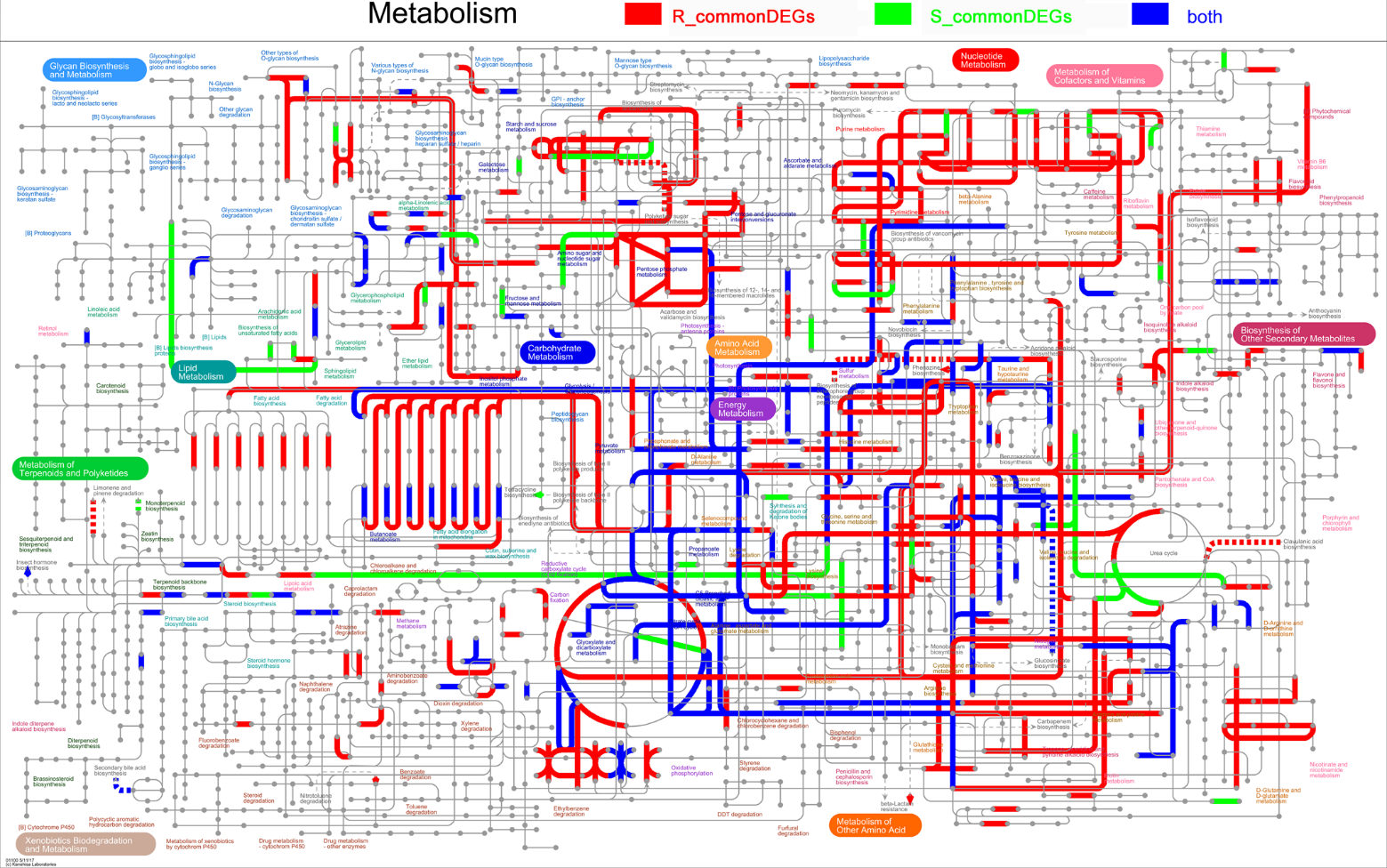


**Supplementary Figure 12.** Metabolism pathway maps of shared DEGs in azole-susceptible SC5314 (shown as S) and azole-resistant F0157 (shown as R) following TPP-C_12_ and TPP-C_14_ treatments. The diagram illustrates pathway annotations across gene sets, where red and light green denote pathways uniquely annotated by genes from distinct sets, blue represents pathways commonly annotated by both sets. The metabolism map encompasses the global metabolic network within the biological system.
